# Treg cells promote decidual vascular remodeling and modulate uterine NK cells in pregnant mice

**DOI:** 10.1172/jci.insight.169836

**Published:** 2024-12-10

**Authors:** Shanna L. Hosking, Lachlan M. Moldenhauer, Ha M. Tran, Hon Y. Chan, Holly M. Groome, Evangeline A.K. Lovell, Ella S. Green, Stephanie E. O’Hara, Claire T. Roberts, Kerrie L. Foyle, Sandra T. Davidge, Sarah A. Robertson, Alison S. Care

**Affiliations:** 1 Robinson Research Institute and School of Biomedicine, The University of Adelaide, Adelaide, South Australia, Australia.; 2 Flinders Health and Medical Research Institute, Flinders University, Adelaide, South Australia, Australia.; 3 Women and Children’s Health Research Institute, Department of Obstetrics and Gynecology, University of Alberta, Edmonton, Alberta, Canada.

**Keywords:** Immunology, Reproductive biology, NK cells, Obstetrics/gynecology, T cells

## Abstract

Regulatory T (Treg) cells are essential for maternal immune tolerance of the fetus and placenta. In preeclampsia, aberrant Treg cell tolerance is implicated, but how Treg cells affect the uterine vascular dysfunction thought to precede placental impairment and maternal vasculopathy is unclear. We used *Foxp3*-diphtheria toxin receptor mice to test the hypothesis that Treg cells are essential regulators of decidual spiral artery adaptation to pregnancy. Transient Treg cell depletion during early placental morphogenesis caused impaired remodeling of decidual spiral arteries, altered uterine artery function, and fewer *Dolichos biflorus* agglutinin^+^ uterine natural killer (uNK) cells, resulting in late-gestation fetal loss and fetal growth restriction. Replacing the Treg cells by transfer from wild-type donors mitigated the impact on uNK cells, vascular remodeling, and fetal loss. RNA sequencing of decidua revealed genes associated with NK cell function and placental extravillous trophoblasts were dysregulated after Treg cell depletion and normalized by Treg cell replacement. These data implicate Treg cells as essential upstream drivers of uterine vascular adaptation to pregnancy, through a mechanism likely involving phenotypic regulation of uNK cells and trophoblast invasion. The findings provide insight into mechanisms linking impaired adaptive immune tolerance and altered spiral artery remodeling, 2 hallmark features of preeclampsia.

## Abstract 2

Regulatory T cells modulate uterine natural killer cells to facilitate uterine spiral artery remodeling, which is crucial for robust placental function and fetal growth

## Graphical abstract

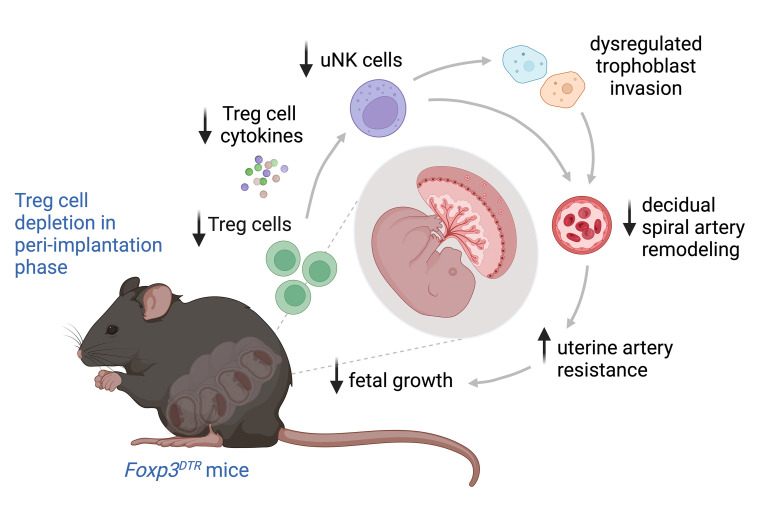

## Introduction

Regulatory T (Treg) cells are a specialized subset of T cells that suppress inflammation and limit deleterious immune responses to self and foreign antigens (1). Treg cells have a pivotal role in immune homeostasis by modulating the proliferation and activity of effector T and B cells and NK cells. Treg cells are essential for pregnancy tolerance; sufficient numbers of functionally competent Treg cells must be present in the uterus for successful embryo implantation and placental development (2). Since pregnancy is an inflammatory state and the fetoplacental unit expresses paternally derived alloantigens (2), the uterine Treg cell pool has important roles in constraining inflammation and preventing aberrant maternal immune reactivity (3–6).

Fewer or functionally impaired Treg cells are a common feature of preeclampsia (7–11), a serious hypertensive disorder that affects 2%–8% of all pregnant women (12, 13). Treg cell deficiency associated with aberrant generation of effector T cells, including pro-inflammatory Th1 and Th17 cells (8, 14), is evident in peripheral blood and uterine decidual tissue in preeclampsia (7–11) and may originate from insufficient priming toward paternal alloantigens or underlying immune dysfunction (15). Although a definitive causal role for Treg cell deficiency in the placental pathophysiology of these conditions is not proven in humans, studies in rodent models (16, 17) indicate biological plausibility, and the significance of paternal factors is consistent with a maladaptive immune response (15, 18).

One candidate mechanism by which Treg cell insufficiency may contribute to defective placentation involves the uterine blood supply (19). Emerging evidence shows Treg cells are essential regulators of systemic vascular homeostasis supporting healthy cardiovascular function in a range of tissues (20, 21). Treg cell insufficiency has been linked to hypertension and aberrant vascular remodeling (22, 23), while treatment interventions to boost Treg cells can reverse cardiovascular dysfunction (24, 25) and mitigate hypertension (24). These considerations raise the prospect that in addition to their immune-regulatory roles, Treg cells might facilitate the vascular adaptations required to accommodate pregnancy — but whether and how this occurs are unknown.

The maternal cardiovascular system must undergo substantial remodeling to support pregnancy and enable optimal placental function. These events commence with local changes to the uterine vasculature during early placental development. Systemic cardiovascular adaptations follow, featuring increased blood volume, enhanced cardiac output, and reduced total peripheral resistance (26). A substantial increase in blood supply to the uterine artery and the placenta supports the increasing nutrient demands of the developing fetus as gestation proceeds (27). Robust fetal growth in late gestation therefore depends on adequate early remodeling and vasodilation of the uterine artery and its network of decidual spiral arteries (27–29).

Decidual spiral arteries are the terminal branch of the main uterine arteries. Their remodeling involves trophoblast invasion of the decidual vessel wall and a loss of the surrounding smooth muscle cells and elastic lamina in women (30), with similar features during placentation in mice (31). Migrating extravillous trophoblast cells invade both the decidual interstitium and the endovascular space within the spiral arteries to facilitate their transformation from high-resistance vessels to low-resistance, high-capacity vessels. These vessels perfuse the placental intervillous space from which exchange of nutrients and oxygen between maternal and fetal circulations occurs (32) to accommodate escalating fetal demand as pregnancy progresses (33).

Immune cells, particularly uterine natural killer (uNK) cells, are critical for decidual spiral artery remodeling (34). uNK cells are an abundant subset of innate lymphoid cells in the decidua (35) that are essential for decidual vascular remodeling in mice (34, 36, 37), and humans (38). They secrete cytokines, such as IFN-γ and VEGFA (35, 37, 39, 40), and enzymes that modulate endothelial cell tight junctions, cause smooth muscle cell apoptosis, and promote trophoblast invasion (34, 41). Elevated inflammatory activity impairs uNK cell function, leading to shallow spiral artery remodeling and placental dysfunction (42, 43).

We previously demonstrated that depletion of Treg cells from pregnant mice causes dysfunction in the main arteries serving the uterus, causing increased resistance and altered regulation of vasoconstriction and affecting systemic blood pressure regulation (19). Additionally, we showed Treg cell deficiency caused by insufficient progesterone signaling elicits impaired decidual spiral artery remodeling (16), building on other studies noting that Treg cell perturbation can be accompanied by uterine vascular changes (2, 19, 44). However, there has been no formal investigation of the specific requirement for Treg cells in remodeling of the decidual spiral arteries or investigation of the cellular mechanisms by which this might occur.

In this study, we test the hypothesis that Treg cells are essential for decidual spiral artery remodeling in early pregnancy, utilizing Forkhead box P3-diphtheria toxin receptor (*Foxp3*^DTR^) transgenic mice (45) to allow acute, transient Treg cell depletion. Here, we report evidence that Treg cell deficiency is a primary cause of poor remodeling of decidual spiral arteries, causing fetal loss and growth restriction in surviving fetuses, and a mechanism involving dysregulation of uNK cells and altered trophoblast invasion is implicated. Our data provide compelling evidence to link Treg cells and uteroplacental vascular remodeling in an interactive network underpinning establishment and progression of healthy pregnancy.

## Results

### Treg cell depletion during the peri-implantation period causes fetal loss and fetal growth restriction.

**Figure 1:**
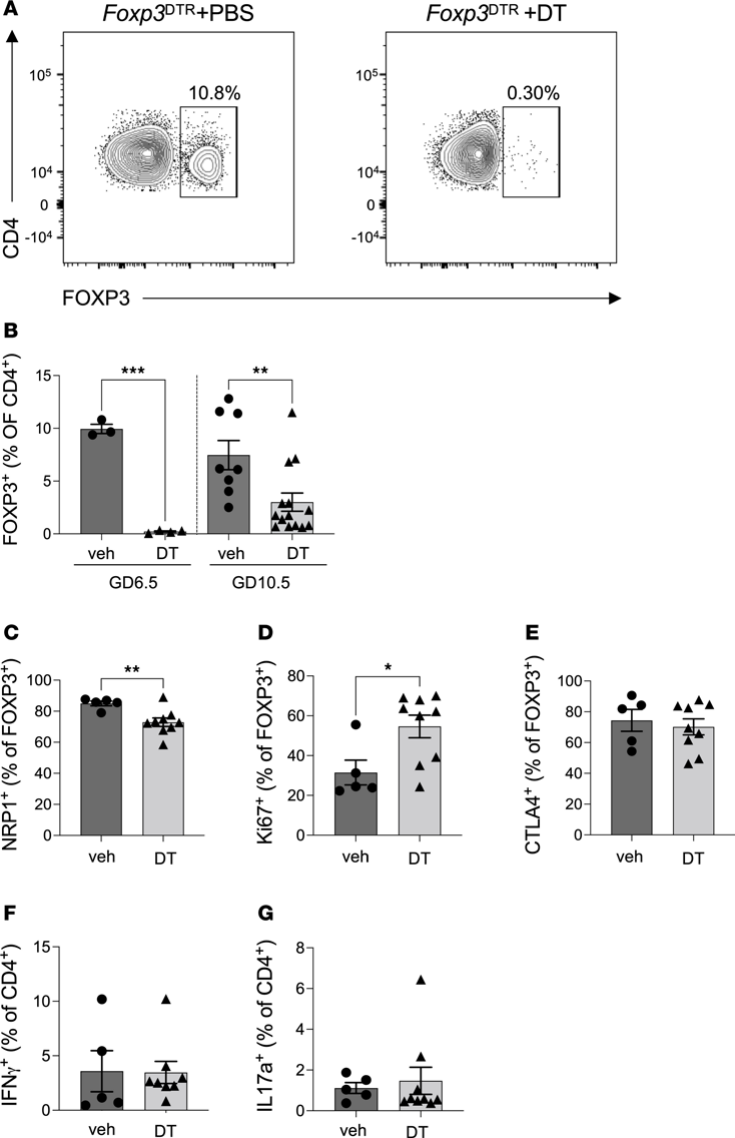
Effect of DT administration to *Foxp3*^DTR^ mice in the peri-implantation period on uDLN Treg cell proportion and phenotype in midgestation. *Foxp3*^DTR^ mice were administered PBS (veh) or DT i.p. on GD3.5 and GD5.5. uDLNs were recovered on GD6.5 or GD10.5, and the proportion and phenotype of CD4^+^FOXP3^+^ Treg cells were evaluated using flow cytometry. (**A**) Representative contour plots show the proportion of CD4^+^FOXP3^+^ Treg cells in the uDLNs on GD6.5 from vehicle-treated (left) and DT-treated (right) *Foxp3*^DTR^ mice. (**B**) The proportions of CD4^+^FOXP3^+^ Treg cells in uDLNs at GD6.5 and GD10.5. Detailed analysis of Treg cells at GD10.5 shows the proportion of Treg cells expressing NRP1 indicating thymic origin (**C**), proliferation marker Ki67 (**D**), and marker of suppressive competence CTLA4 (**E**). The proportion of IFN-γ^+^CD4^+^FOXP3^–^ (Th1 cells; **F**) and IL-17a^+^CD4^+^FOXP3^–^ (Th17 cells; **G**) were measured. *N* = 3–14 mice per group. Data are mean ± SEM. Data points are values from individual dams. Analysis was by 2-tailed *t* test or Mann-Whitney *U* test depending on normality of data distribution for data in **C**–**G**. Data in **B** were analyzed using a 1-way ANOVA comparing samples within the same gestational day. *P < 0.05; **P < 0.01; ***P < 0.001.

To evaluate the physiological significance of Treg cells in the peri-implantation phase for pregnancy progression, we administered DT on gestational day (GD)3.5 and 5.5 to selectively and transiently deplete the Treg cell pool, as previously described (19). In *Foxp3*^DTR^ mice treated with DT, Treg cells (measured as a proportion of CD4^+^ T cells) were reduced by at least 97% in the uterine-draining, para-aortic lymph nodes (uDLNs) by GD6.5, 24 hours posttreatment. This site is where Treg cells normally proliferate before recruitment into the uterus (46) (Figure 1, A and B). In contrast, Treg cells in vehicle-treated control *Foxp3*^DTR^ mice remained unchanged. Analysis at the critical midgestation time point of GD10.5, when the mouse placenta is mature and placental access to the maternal blood supply is complete (30, 47), showed partial repopulation of Treg cells in the uDLNs, although their proportion remained approximately 59% less than in control mice (Figure 1B). The majority of Treg cells in the uDLNs in midgestation were thymic derived, as indicated by neuropilin 1 (NRP1) expression (48), regardless of earlier Treg cell depletion (Figure 1C). Repopulating Treg cells more commonly expressed the proliferation marker Ki67 (Figure 1D), while similar proportions expressed cytotoxic T lymphocyte antigen 4 (CTLA4), a marker of suppressive competence (49) (Figure 1E). Treg cell deficiency did not cause an increase in CD4^+^ T cell expression of IFN-γ or IL-17a (Figure 1, F and G). Comparable effects were seen in the spleen, consistent with a systemic impact of DT on Treg cells in *Foxp3*^DTR^ mice (Supplemental Figure 1; supplemental material available online with this article; https://doi.org/10.1172/jci.insight.169836DS1). Recovery was faster in the spleen, such that on GD10.5 Treg cells were not different in proportion or phenotype between DT-treated and control mice (Supplemental Figure 1, A–D), and IFN-γ and IL-17a expression was unchanged (Supplemental Figure 1, E and F).

**Figure 2:**
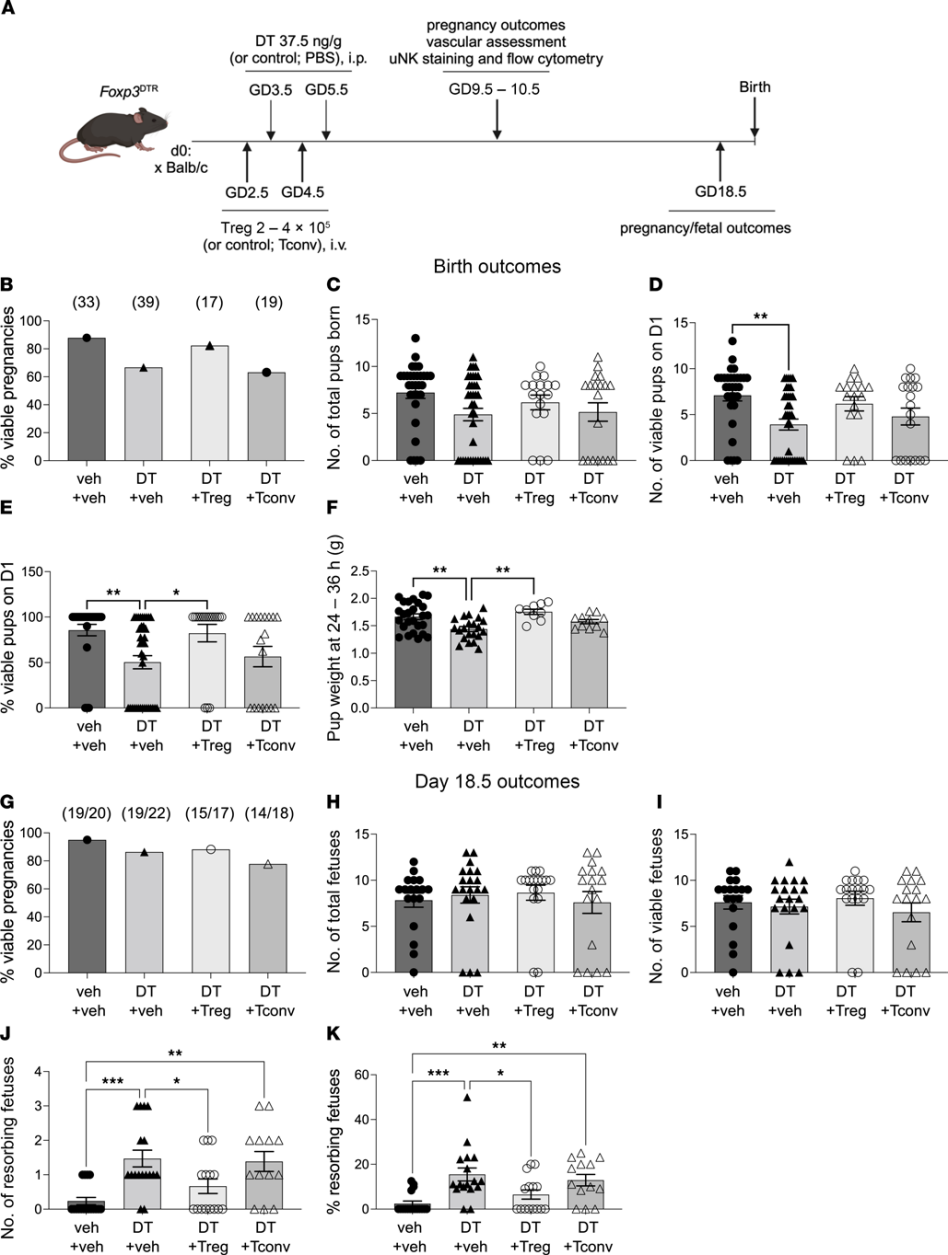
Treg cell depletion in the peri-implantation period results in reduced litter size at birth and increased fetal loss in late gestation and is mitigated by Treg cell transfer. *Foxp3*^DTR^ mice were administered PBS (veh) or DT i.p. on GD3.5 and GD5.5, and then pregnancy outcomes were assessed either at birth (**B**–**F**) or on GD18.5 (**G**–**J**). Some mice also received WT Treg cells or Tconv cells on GD2.5 and GD4.5. (**A**) Schematic diagram showing the treatment and analysis protocol (created with BioRender.com). (**B**) Proportion of mated mice that delivered at least 1 viable pup at birth. (**C**) Total number of pups at birth per dam. (**D**) Number of viable pups within 24 hours of birth per dam. (**E**) Proportion of viable pups within 24 hours of birth per dam. (**F**) Pup weight at 24–36 hours after birth. (**G**) The proportion of dams carrying a viable pregnancy at GD18.5 (defined as at least 1 viable implantation site). (**H**) Number of total fetuses per dam at GD18.5. (**I**) Number of viable fetuses per dam at GD18.5. (**J**) Number and (**K**) proportion of resorbing fetuses per dam at GD18.5. Numbers of mice (**B**) and dams (**G**) in each group are shown in parentheses. Data are mean ± SEM. Data points are values from individual dams. Analysis was by χ^2^ test (**B** and **G**) or 1-way ANOVA with 2-tailed post hoc *t* test (**C**–**F** and **H**–**K**). *P < 0.05; **P < 0.01; ***P < 0.001.

To evaluate the impact of peri-implantation pregnancy Treg cell depletion on pregnancy success, *Foxp3*^DTR^ mice were given DT or vehicle control and birth outcomes were recorded. Additional groups of DT-treated *Foxp3*^DTR^ mice were administered wild-type (WT) Treg cells (CD4^+^CD25^+^ cells) that do not express human DTR and are refractive to DT-induced depletion (45) or conventional CD4^+^ T cells (Tconv cells; CD4^+^CD25^–^ cells), on GD2.5 and GD4.5. Transferred cells were prepared from the spleens and lymph nodes of pregnant donor mice to ensure hormone and antigen priming, which are important for Treg cell function in pregnancy (50). A subset of dams were allowed to give birth, and birth outcomes were evaluated. For another subset, pregnancy outcomes were evaluated in late gestation (Figure 2A).

Compared with control dams administered vehicle, Treg cell depletion compromised pup viability at birth (Figure 2, D and E) and reduced pup weight (Figure 2F) but had no impact on the proportion of dams with viable pregnancies (defined as 1 or more viable pup) or total pups born (Figure 2, B and C). Pretreatment with WT Treg cells in DT-treated *Foxp3*^DTR^ mice prevented the shift in pregnancy success, litter size, and birth weight, while Tconv cells did not confer the same improvement (Figure 2, D–F).

The poor pregnancy outcomes after peri-implantation Treg cell depletion prompted us to evaluate late-gestation fetal and placental parameters. In DT-treated and vehicle control *Foxp3*^DTR^ mice autopsied at GD18.5, the rate of viable pregnancy (defined as at least 1 viable fetus) and total or viable implantation sites per dam (Figure 2, G–I) were unchanged. However, DT treatment caused an increase in both the number and the proportion of overt fetal losses (resorptions) per dam compared with control mice (Figure 2, J and K), indicating fetal loss occurred after midgestation. Pretreatment with WT Treg cells mitigated Treg cell deficiency (Figure 2, J and K), such that resorptions were not different compared to control mice. In contrast, Tconv cells did not attenuate the effects of Treg cell deficiency (Figure 2, J and K).

**Figure 3:**
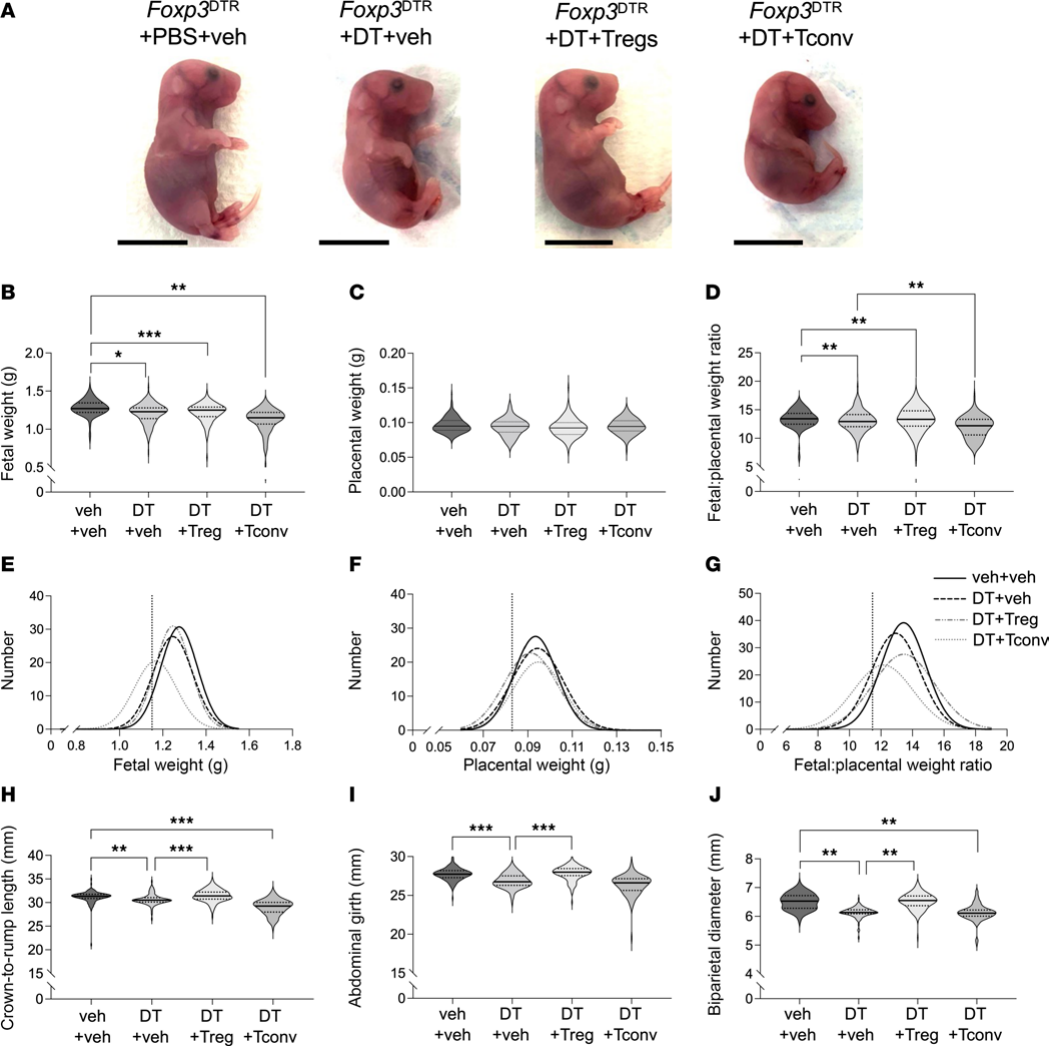
Treg cell depletion in the peri-implantation period causes fetal growth restriction that is mitigated by Treg cell transfer. *Foxp3*^DTR^ mice were administered PBS (veh) or DT i.p. on GD3.5 and GD5.5, and then fetal and placental development were assessed on GD18.5. Some mice also received WT Treg cells or Tconv cells on GD2.5 and GD4.5. (**A**) Representative images of fetuses, (**B**) fetal weight, (**C**) placental weight, and (**D**) fetal/placental weight ratio. The distribution of (**E**) fetal weights, (**F**) placental weights, and (**G**) fetal/placental weight ratio. (**H**) Fetal crown-to-rump length, (**I**) abdominal girth, and (**J**) biparietal diameter. Vertical dashed line represents the 10th centile of the curve (1.15, 0.08, and 11.45 g in **E**–**G**, respectively). *N* = 2–11 fetuses from 14–19 dams per group. Fetal and placental data are shown as violin plots with median and quartile values marked. Analysis was by mixed-model ANOVA with mother as subject and litter size as covariate. *P < 0.05; **P < 0.01; ***P < 0.001. Scale bar = 1 cm.

Surviving fetuses from *Foxp3*^DTR^ mice treated with DT to elicit Treg cell depletion were growth restricted on GD18.5, compared with vehicle-treated controls. Fetal weight was reduced by 3.1%, crown-to-rump length by 1.9%, abdominal girth by 3.4%, and biparietal diameter by 5.0% (Figure 3, A, B, and H–J). Transfer of WT Treg cells to DT-treated mice did not correct late-gestation fetal weight (Figure 3B) but did mitigate other parameters of fetal growth (Figure 3, H–J). In contrast, Tconv cells further reduced fetal weight, crown-to-rump length, and biparietal diameter (Figure 3, B, H, and J). When fetal weight distribution was considered (Figure 3E), 27% were below the 10th percentile in DT-treated mice, compared with 21% in dams given DT and Treg cells and 46% in dams given DT and Tconv cells.

There was no impact of Treg cell depletion from *Foxp3*^DTR^ mice, or exogenous Treg cell or Tconv cell administration, on placental weight (Figure 3, C and F). However, Treg cell depletion reduced the fetal/placental weight ratio, a surrogate measure of placental efficiency (Figure 3, D and G). This was unchanged by Treg cell or Tconv transfer (Figure 3D). This indicates that as well as fetal loss, Treg cell depletion impaired placental efficiency in surviving fetuses, in turn affecting fetal growth. Together, these data show that Treg cell deficiency, and not off-target effects of DT treatment, are responsible for fetal loss and fetal growth impairment. Consistent with this interpretation, no effect of DT on fetal survival, fetal weight, or growth parameters was seen in WT C57BL/6 mice given DT on GD3.5 and 5.5, compared to mice given vehicle (Supplemental Figure 2).

To further investigate the impacts of Treg cell depletion, placental structure was assessed in late gestation (Supplemental Figure 3, A–D). There was no effect of Treg cell depletion on placental structure at GD18.5 as indicated by area of junctional zone (JZ) or labyrinth zone, the tissues responsible for endocrine function and nutrient transfer, respectively (Supplemental Figure 3, E–I). The proportion of glycogen cells in the JZ was increased following DT treatment to elicit Treg cell depletion compared with control mice. This was prevented by treatment with Treg cells but not Tconv cells (Supplemental Figure 3J).

### Treg cell depletion disrupts uterine vascular function and decidual spiral artery remodeling.

We next investigated the impact of Treg cell depletion on the uterine vasculature in midgestation, as this is a critical determinant of placental maturation and capacity to support fetal survival and growth. At GD10.5, *Foxp3*^DTR^ dams given DT to elicit Treg cell depletion had a similar rate of viable pregnancy, number of total and viable implantation sites per dam, and proportion and number of abnormal implantation sites showing early signs of resorption compared with vehicle-treated control *Foxp3*^DTR^ dams. Transfer of Treg cells or Tconv cells did not change these parameters (Supplemental Figure 4, A–I).

**Figure 4:**
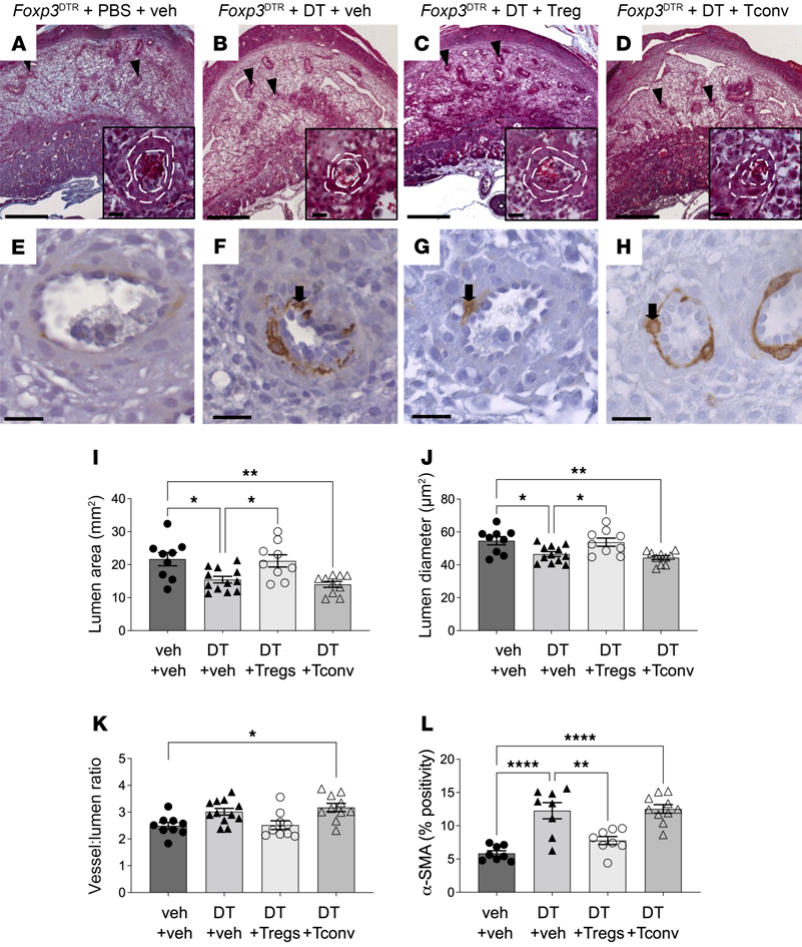
Treg cell depletion impairs spiral artery remodeling in midgestation. Pregnant *Foxp3*^DTR^ mice were administered PBS (veh) or DT i.p. on GD3.5 and GD5.5, and then tissues were collected on GD10.5. Some mice also received WT Treg cells or Tconv cells on GD2.5 and GD4.5, and then tissues were collected on GD10.5. Representative images of midsagittal sections of uterus stained with Masson’s trichrome (**A**–**D**) or to detect α-SMA (**E**–**H**). Black arrowheads indicate spiral arteries, black arrows indicate α-SMA^+^ cells. Parameters including (**I**) average lumen area of spiral arteries, (**J**) lumen diameter, (**K**) vessel/lumen area ratio, and (**L**) proportion of decidual cells positive for α-SMA are shown. *N* = 2 implantation sites per dam from 8–12 dams per group. Data are mean ± SEM. Data points are average values for individual dams. Analysis was by 1-way ANOVA with 2-tailed post hoc *t* test. *P < 0.05; **P < 0.01; ****P < 0.0001. Scale bars, **A**–**D** = 1 mm; insets = 50 μm; **E**–**H** = 50 μm.

When decidual spiral artery remodeling was quantified stereologically in midsagittal sections of implantation sites on GD10.5, impaired spiral artery remodeling was seen after Treg cell depletion (Figure 4, A–D). Decidual spiral artery lumen area and lumen diameter were reduced by 29% and 15%, respectively, compared with control (Figure 4, A, B, I, and J). There was a trend toward an increased relative wall thickness in decidual spiral arteries (vessel/lumen ratio) after Treg cell depletion (Figure 4K; *P* = 0.08). Transfer of WT Treg cells normalized decidual spiral artery remodeling (Figure 4, C, I, and J), but Tconv cells did not (Figure 4, D, I, and J), and increased the relative wall thickness of vessels, compared with control mice (Figure 4K).

Smooth muscle cells are normally lost from the arterial vascular media during the spiral artery remodeling process, and α–smooth muscle actin (α-SMA) retention indicates impaired remodeling (51). Depletion of Treg cells from *Foxp3*^DTR^ mice resulted in a 2-fold increase of α-SMA expression compared with control *Foxp3*^DTR^ mice (Figure 4, E, F, and L). Loss of α-SMA was improved by transfer of Treg cells (Figure 4, G and L) but not Tconv cells (Figure 4, H and L).

**Figure 5:**
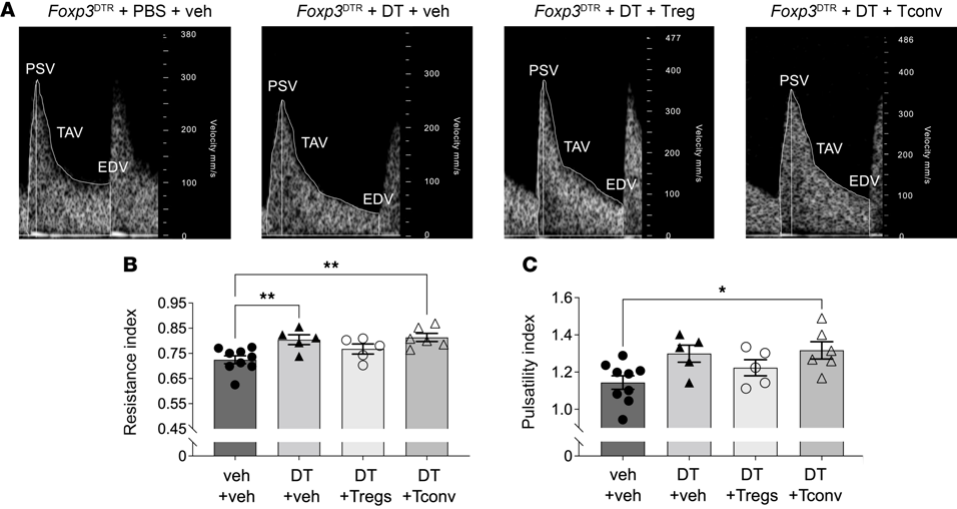
Uterine artery resistance in midgestation is increased by Treg cell depletion and mitigated by Treg cell replacement. Pregnant *Foxp3*^DTR^ mice were administered PBS (veh) or DT i.p. on GD3.5 and GD5.5, and then tissues were collected on GD10.5. Some mice also received WT Treg cells or Tconv cells on GD2.5 and GD4.5. Measurements were taken on GD9.5. (**A**) Representative waveforms of uterine arteries. (**B**) Resistance index and (**C**) pulsatility index were calculated. *N* = 5–8 dams per group. Data are mean ± SEM. Data points are average values for individual dams. Analysis was by 1-way ANOVA with 2-tailed post hoc *t* test. EDV, end-diastolic velocity; PSV, peak systolic velocity; TAV, time-averaged velocity. *P < 0.05; **P < 0.01.

To determine the impact of Treg cell perturbation in the peri-implantation phase on function of the main uterine arteries in midgestation, we used ultrasound biomicroscopy on GD9.5. Treg cell depletion was found to increase uterine artery resistance index by 11.0%, indicating increased resistance to blood flow (Figure 5, A and B). Administration of Treg cells, but not Tconv cells, mitigated the increase in uterine artery resistance (Figure 5B). Uterine artery pulsatility index, another measure of resistance, showed an increasing trend following Treg cell depletion (*P* = 0.06; Figure 5C) that was partially improved by Treg cell transfer but not Tconv cells (Figure 5C). These findings build upon our previous report of altered uterine vascular parameters after Treg cell depletion (19). The effect of Treg cell depletion on uterine artery function was limited to midpregnancy; when uterine artery and umbilical artery function were measured on GD17.5, there was no impact of Treg cell depletion or treatment with Treg or Tconv cells (Supplemental Table 1).

### Treg cell depletion modifies uNK cells, key regulators of spiral artery remodeling.

**Figure 6:**
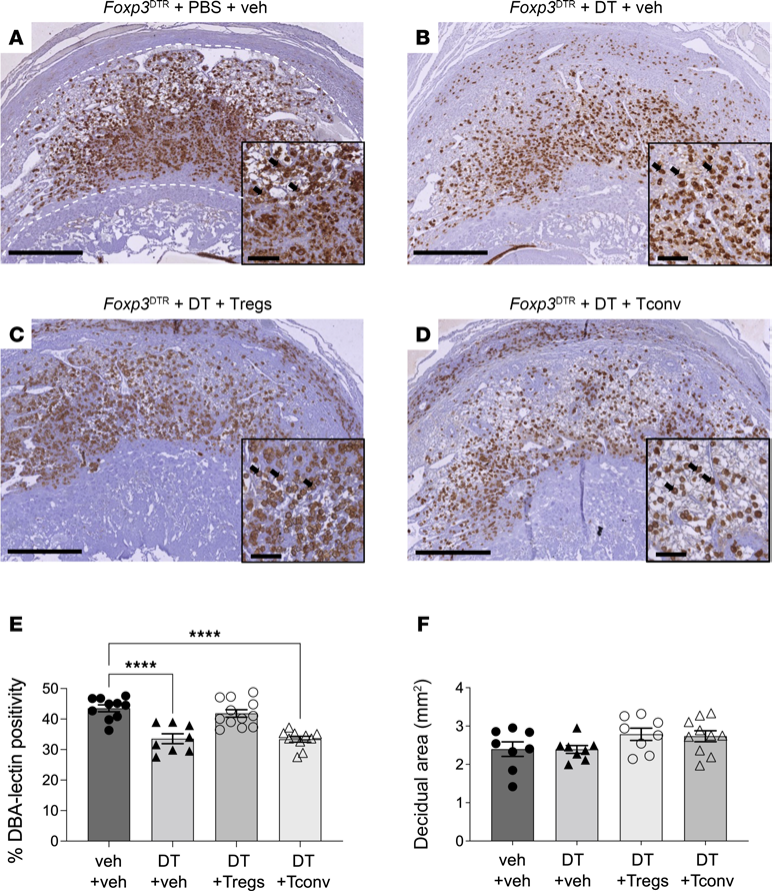
Treg cell depletion causes a reduction in DBA^+^ uNK cells in midgestation that is mitigated by Treg cell transfer. Pregnant *Foxp3*^DTR^ mice were administered PBS (veh) or DT i.p. on GD3.5 and GD5.5, and then tissues were collected on GD10.5. Some mice also received WT Treg cells or Tconv cells on GD2.5 and GD4.5. Tissues were collected on GD10.5. (**A**–**D**) Decidual tissue sections were labeled with biotinylated DBA-lectin to detect the DBA^+^ subset of uNK cells that predominates in pregnancy (brown stain, arrows). (**E**) The percentage positivity for DBA^+^ uNK cells was quantified. (**F**) The decidual region of each section (marked, dotted line in **A**) was identified and measured. *N* = 2 implantation sites per dam, 8–12 dams per group. Data are mean ± SEM. Data points are average values for individual dams. Analysis was by 1-way ANOVA with 2-tailed post hoc *t* test. ****P < 0.0001. Scale bars: 500 μm; insets = 100 μm.

Given the central role of uNK cells in spiral artery remodeling, we next investigated whether uNK cells contribute to the mechanism by which Treg cell deficiency causes remodeling impairment. Initially, uNK cell abundance was assessed by histological staining with *Dolichos biflorus* agglutinin (DBA) lectin to identify uNK cells in midsagittal sections of implantation sites. DBA reacts with *N*-acetylgalactosamine (GalNAc) residues present on the cell surface and in granules of uNK cells (52). DBA^+^ uNK cells, which predominate in pregnancy, produce factors including VEGFA that facilitate spiral artery remodeling by promoting migration of extravillous trophoblasts through the decidua (40), while DBA^–^ uNK cells produce IFN-γ (35, 40). Compared with vehicle-treated control *Foxp3*^DTR^ mice, Treg cell–depleted *Foxp3*^DTR^ mice had substantially less DBA staining in the decidua on GD10.5 (Figure 6, A, B, and E). DBA^+^ uNK cell abundance was normalized by Treg cell transfer but not by Tconv cells (Figure 6, A–E). Notably, the total midsagittal area of decidual tissue was not affected by Treg cell depletion, indicating the vascular and uNK cell changes were unlikely to be due to defective decidualization (Figure 6F).

To further understand the impact of Treg cell depletion, uNK cells were investigated by flow cytometry. uNK cells can be classified into 3 subsets with distinguishing surface phenotypes with potential for distinct roles in angiogenesis and vascular remodeling (53). All 3 subsets express the NK lineage-defining surface marker NK1.1 (54) and acquire NKp46 (also termed natural cytotoxicity receptor 1) upon maturation in the decidua (55–58). The uNK cell subsets include (a) tissue-resident NK (trNK) cells, defined as CD45^+^NK1.1^+^CD11B^lo/–^NKp46^+^CD49a^+^EOMES^+^ cells; (b) conventional NK (cNK) cells. defined as CD45^+^NK1.1^+^CD11B^lo/–^NKp46^+^CD49a^–^ cells, and (c) group 1 innate lymphoid (ILC1) cells, defined as CD45^+^NK1.1^+^CD11B^lo/–^NKp46^+^CD49a^+^EOMES^–^ cells. cNK cells are the primary source of IFN-γ (58) while trNK cells produce VEGFA. When trNK, cNK, and ILC1 cells were analyzed in decidua recovered on GD10.5 from vehicle- or DT-treated *Foxp3*^DTR^ mice, as expected, the largest population was trNK cells, followed by cNK and then ILC1 cells (Supplemental Figure 5, A–E) (58). Treg cell depletion did not change the proportion of NK1.1^+^ uNK cells categorized as cNK, ILC1, or trNK compared to vehicle-treated control mice (Supplemental Figure 5, C–E). Similarly, no change was seen in the proportion of NK1.1^+^ or NKp46^+^ NK cells among non-T, non-B, or total CD3^+^ T cells (Supplemental Figure 5, F–J).

### Transcriptomic analysis shows canonical NK cell and T cell pathways are disrupted by Treg cell depletion and restored by Treg cell replacement.

**Figure 7:**
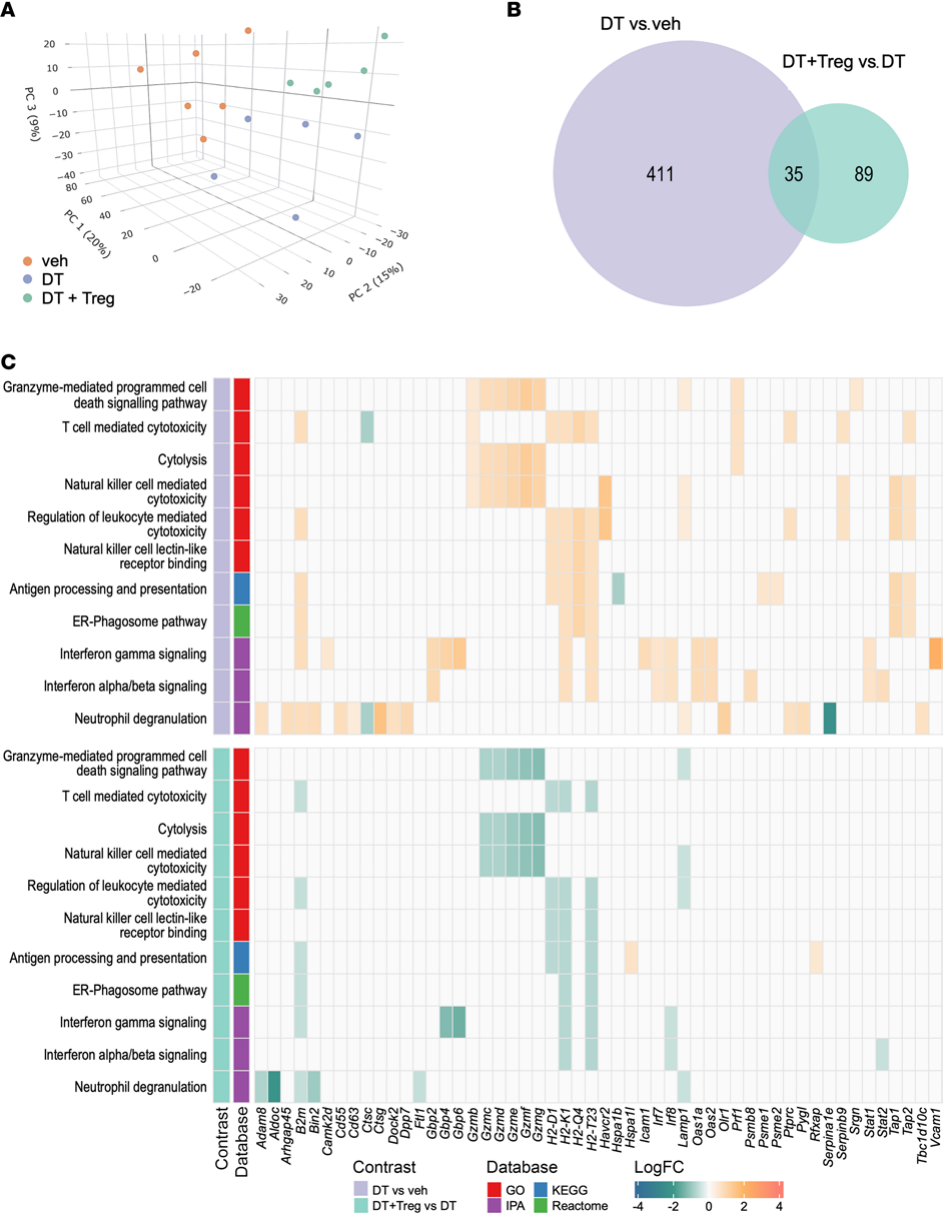
Treg cell depletion causes a perturbation in decidual transcription profile in midgestation that is mitigated by Treg cell transfer. Pregnant *Foxp3*^DTR^ mice were administered PBS (veh; *n* = 6) or DT (*n* = 5) i.p. on GD3.5 and GD5.5, and decidual tissue was collected on GD10.5. Some mice (*n* = 5) also received WT Treg cells on GD2.5 and GD4.5. (**A**) PCA of filtered genes, illustrating gene expression patterns in individual samples. (**B**) The number of DEGs (FDR < 0.1) that overlap between DT-treated mice compared with PBS vehicle control– (purple) and DT+Treg–treated mice compared with DT-treated mice (green). (**C**) Functional heatmap of DEGs (FDR < 0.1) and their relationship to enriched terms/pathways identified using Gene Ontology (GO, FDR < 0.05), Kyoto Encyclopedia of Genes and Genomes (KEGG, FDR < 0.2), Reactome (FDR < 0.1), and Ingenuity Pathway Analysis (IPA, *P* < 0.05) databases.

The data indicating fewer DBA^+^ uNK cells compelled us to investigate the impact of Treg cell depletion on gene transcription in the decidua following Treg cell depletion. We performed RNA sequencing on decidual tissue collected at GD10.5 from vehicle-treated mice (veh), DT-treated *Foxp3*^DTR^ mice (DT), and DT-treated *Foxp3*^DTR^ mice given WT Treg cells (DT+Treg; *n* = 5–6 samples per group; see Supplemental Table 2 for RNA-sequencing metrics). After removal of non- and lowly expressed genes, 14,748 genes were trimmed mean of M-values (TMM) normalized, and subsequent dimensionality reduction via principal component analysis (PCA) identified 3 distinct clusters corresponding to the 3 treatment groups (Figure 7A).

Transcriptomic profiling identified 446 differentially expressed genes (DEGs) following Treg depletion compared with the vehicle-treated group (DT vs. veh), including 192 upregulated and 254 downregulated genes (all DEGs detected are listed in the Supporting Data Values file, and the top 50 up- and downregulated genes are listed in Supplemental Tables 3 and 4). In mice that received Treg cells as well as DT (DT+Treg group), the majority of the 446 DEGs were unchanged, and only 35 of the 446 DEGs were differentially expressed (Figure 7B), indicating partial normalization of the transcriptional changes.

To understand their functional significance, overrepresentation analysis of the DEGs from these comparisons was performed using Gene Ontology (GO) (59), Kyoto Encyclopedia of Genes and Genomes (KEGG) (60), Reactome (61), and Ingenuity Pathway Analysis (IPA, QIAGEN) databases (Supplemental Figures 6–8). Pathways regulated by Treg depletion during the peri-implantation phase predominantly reflected immune effector responses, particularly those involving NK cell and T cell activation and cytotoxicity. Notably, GO analysis identified granzyme-mediated programmed cell death signaling, T cell mediated cytotoxicity, NK cell mediated cytotoxicity, and NK cell lectin-like receptor binding pathways (Supplemental Figure 6), while KEGG analysis identified pathways including graft versus host disease, allograft rejection, and natural killer cell-mediated cytotoxicity (Supplemental Figure 7). IPA revealed interferon gamma signaling and interferon alpha/beta signaling were among the top canonical pathways (Supplemental Figure 8) and biological functions invoking lymphocyte cytotoxicity (Supplemental Figure 9). The logFCs of DEGs linked with selected pathways associated with NK cell and T cell activation are shown in Figure 7C.

Strikingly, transfer of WT Treg cells to DT-treated *Foxp3*^DTR^ mice substantially normalized most of the DEGs (Supplemental Tables 3 and 4), including many of the DEGs annotated to NK and T cell activation pathways (Figure 7C). Among the effector genes upregulated by Treg depletion and downregulated by Treg cell replacement were genes encoding granzyme B (*Gzmb*) and perforin pore-forming proteins (*Prf1*) that mediate lymphocyte cytotoxicity, as well as granzymes associated with remodeling activity *Gzmc*, *Gzmd*, *Gzme*, *Gzmf*, and *Gzmg* (62–64). Certain NK cell–associated transcription factors were upregulated after Treg cell depletion, notably *Eomes*, *Id2*, and *Runx3*, which control aspects of NK cell maturation and function. Many genes involved in the interferon response pathway, a key program associated with activation of cNK and ILC1 cells, were also upregulated, as were genes *Il15ra*, *Il2rb*, and *Il2rg* encoding the 3 components of the trimeric IL-15 receptor (IL-15Rα, IL-15Rβ, and IL-2Rγ) that mediates uNK cell responsiveness to the uNK cell–activating cytokine IL-15.

**Figure 8:**
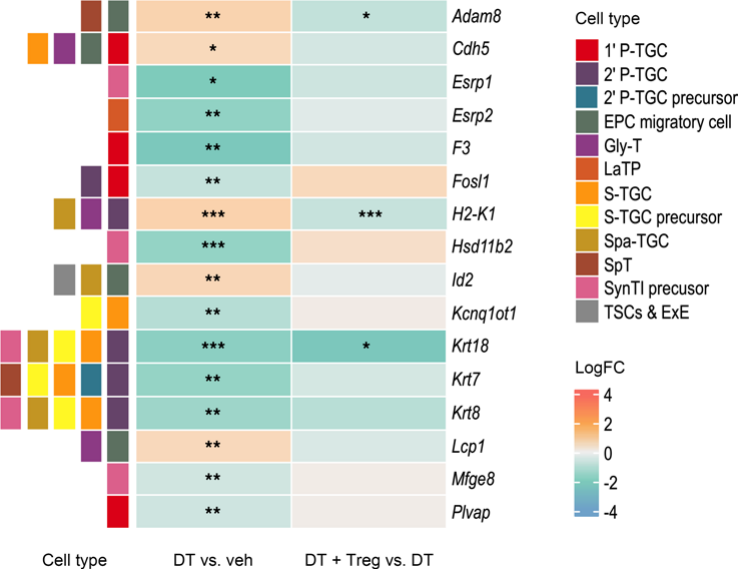
Treg cell depletion causes a perturbation in decidual trophoblast genes in midgestation that is mitigated by Treg cell transfer. Pregnant *Foxp3*^DTR^ mice were administered PBS (veh; *n* = 6) or DT (*n* = 5) i.p. on GD3.5 and GD5.5, and decidual tissue was collected on GD10.5. Some mice (*n* = 5) also received WT Treg cells on GD2.5 and GD4.5. Functional heatmap of DEGs (FDR < 0.1) identified as indicative of altered extravillous trophoblasts on the basis of both (a) reported expression in specific mouse trophoblast cell types (color coded, LHS) (extracted from published single-cell RNA-sequencing data) (66) and (b) expression in mouse placenta but not mouse uterus, according to Mouse Genomics Informatics database (see Methods for details). Cell labels indicate the FDR-adjusted *P* value (FDR) of DEGs present in the RNA-sequencing dataset. *FDR < 0.1; **FDR < 0.05; ***FDR < 0.01. 1’ P-TGC, primary parietal trophoblast giant cell; 2’ P-TGC, secondary parietal trophoblast giant cells; EPC, ectoplacental cone; Gly-T, glycogen trophoblast cells; LaTP, labyrinthine trophoblast; S-TGC, sinusoid trophoblast giant cell; Spa-TGC, spiral artery-associated trophoblast giant cell; SpT, spongiotrophoblast cell; SynT1, multinucleated syncytiotrophoblast cells; TSC, trophoblast stem cell; ExE, extraembryonic ectoderm.

An extensive array of genes known to be expressed by placental trophoblasts were also present among the DEGs (Supplemental Figure 10). A subset of these trophoblast genes selected on the basis of their absence in uterine cells (indicating trophoblast specificity) (65) included *Cdh5*, *Lcp1*, and *Olr1*, all of which are associated with placental glycogen cells (65) (Figure 8 and Supplemental Figure 10). Others including *Krt7*, *Krt8*, *Psck6*, *Cited4*, and *Hsd11b2* expressed by various extravillous trophoblast lineages (66) were downregulated in decidual tissue after Treg cell depletion (Figure 8). These trophoblast genes were largely normalized after Treg cell replacement (Figure 8 and Supplemental Figure 10).

Notably, there was no evidence of altered expression of hallmark decidualization genes *Prl*, *Igfbp1*, *Foxo1*, *Fstl1*, *Bmp2*, or *Ptgs2* following Treg depletion as might be expected if decidual development or function were compromised (Supporting Data Values file). These observations clearly point to the significance of Treg cells in constraining uNK cell activation pathways under normal conditions and strongly suggest a role for inappropriately activated uNK cells and altered trophoblast cell invasion or survival in the observed vascular remodeling and fetal loss seen after Treg cell depletion.

### Treg cell–derived antiinflammatory cytokines are implicated in uNK cell regulation.

Upstream regulator analysis in IPA was performed to identify factors implicated as potential regulators of the Treg cell depletion–induced decidual gene transcription changes. The predicted upstream regulators included 93 candidate immune-regulatory cytokines, cytokine receptors, and transcription factors. Around half of these overlapped with genes detected in decidual tissue, and some were DEGs regulated by Treg cell depletion and replacement (Supplemental Figure 11). Notably, the predicted regulators included 2 factors known to be released by Treg cells with potential to modulate uNK cell function, IL-10 (activation *Z*-score = 2.1) and EBI3 (a subunit of IL-35; activation *Z*-score = 2.6). IL-35 is an immunosuppressive cytokine composed of 2 chains, IL-12α (p35) and EBI3 (67), that is produced exclusively by Treg cells to modulate the function of NK cells (54, 68). The upstream regulator analysis also implicated IFN-γ (activation *Z*-score = 6.1).

**Figure 9:**
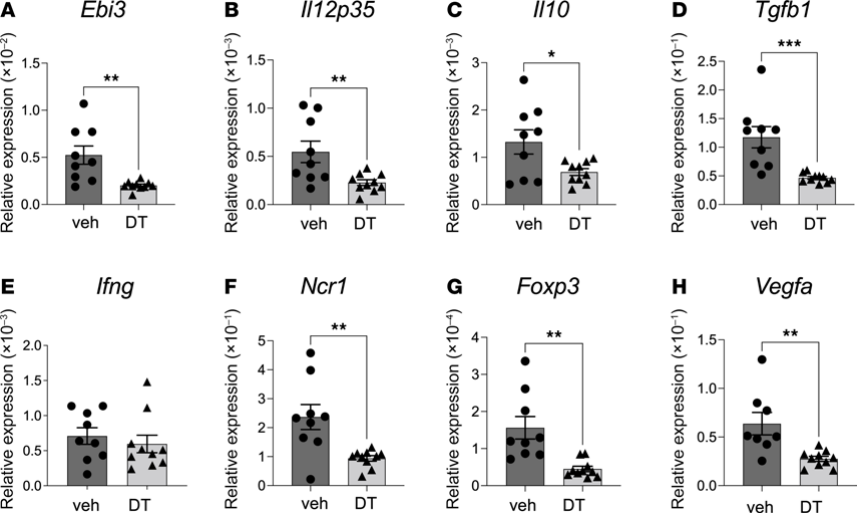
Peri-implantation Treg cell depletion elicits reduction of antiinflammatory decidual gene transcripts. Pregnant *Foxp3*^DTR^ mice were given PBS (veh) or DT on GD3.5 and GD5.5, and then tissues were collected on GD10.5. (**A**) *Ebi3*, (**B**) *Il12p35* genes encoding IL-35, (**C**) *Il10*, (**D**) *Tgfb1*, (**E**) *Ifng*, (**F**) *Ncr1* encoding NKp46, (**G**) *Foxp3* encoding FOXP3 transcription factor necessary for Treg cell development, and (**H**) *Vegfa* were quantified by qPCR. *N* = 9–10 dams per group. Data are presented as mean ± SEM. Each data point represents the average of 2 decidua per dam. Analysis was by unpaired 2-tailed *t* test. *P < 0.05; **P < 0.01; ***P < 0.001.

Because these regulators were not detected by RNA sequencing, presumably due to insufficient sequencing depth, we used quantitative PCR (qPCR) to measure decidual expression of *Il12p35*, *Ebi3*, and *Il10*, as well as *Tgfb1*, another key Treg cell–derived immune regulatory cytokine (67), plus uNK cell factors *Ifng* and *Vegf* involved in vascular remodeling (35, 37, 51). In the same GD10.5 decidual samples utilized in the RNA-sequencing analysis, peri-implantation Treg cell depletion resulted in a 62% reduction in *Ebi3* expression and a 58% downregulation of *Il12p35* expression, compared with control *Foxp3*^DTR^ mice (Figure 9, A and B). Similarly, *Il10* was reduced by 48% and *Tgfb1* was reduced by 60% after Treg cell depletion (Figure 9, C and D). In contrast, decidual *Ifng* was not impacted (Figure 9E), but *Vegfa* expression was reduced by 57% compared with control mice (Figure 9H). Decidual expression of *Foxp3* mRNA encoding the Treg cell transcription factor FOXP3 was reduced by 71% after earlier Treg cell depletion (Figure 9G), while *Ncr1* encoding the uNK cell maturation marker NKp46 was reduced by 60% (Figure 9F), consistent with the histological finding of fewer uNK cells in decidual tissues.

## Discussion

There is compelling evidence that dysregulation in the number and/or function of Treg cells contributes to the pathophysiological origin of preeclampsia and related pregnancy complications (69, 70), but how Treg cells contribute to the uterine and placental dysfunction underpinning these conditions remains unclear. In this study, *Foxp3*^DTR^ mice were used to selectively and transiently deplete Treg cells to investigate their role in the critical process of decidual spiral artery remodeling required in early pregnancy for robust placental development and function. Here, we demonstrate that Treg cell depletion causes impaired spiral artery remodeling, resulting in fetal loss and fetal growth restriction. Treg cell depletion was associated with an altered transcriptional profile in the decidua that along with histochemical analysis implicates dysregulation of uNK cells and extravillous trophoblasts in midgestation. In particular, depletion of Treg cells caused upregulation of genes involved in NK cell IFN signaling and cytotoxic function, notably granzyme-mediated programmed cell death signaling. Treg cell replacement experiments largely rescued the reproductive phenotype and reversed the gene expression changes, verifying that Treg cells affect decidual spiral artery remodeling and supporting the inference of a mechanism involving Treg cell modulation of uNK cell functional status. This requirement for Treg cells provides insight into immune regulation of uterine vascular remodeling, extending our previous demonstration of a role for Treg cells in regulating the main uterine arteries in pregnancy (19). If a similar effect of Treg cells on uterine vascular remodeling occurs in women, Treg cell deficiency would act to constrain the placental vascular supply, contributing to development of preeclampsia and other pregnancy disorders characterized by impaired placentation, including recurrent miscarriage, fetal growth restriction, and spontaneous preterm birth (2).

After embryo implantation in mice, the uterine lining undergoes decidualization, and extravillous trophoblasts invade the decidua both interstitially and endovascularly, to progress remodeling of the decidual spiral arteries and enable placental growth (71, 72) and full access to the maternal blood supply by GD10.5 (30, 47). Remodeling is a process that involves displacement of endothelial cells and smooth muscle cells from the spiral artery wall, converting them to flaccid conduits. uNK cells are the main maternal immune cells present in the decidua at the time of implantation (34, 41). They engage with trophoblasts in clusters around spiral arteries and are paramount in facilitating the remodeling process by secreting cytokines and proteases (37, 39). Treg cells, macrophages, and other immune cells are positioned within the decidual stroma with the potential to interact with uNK cells, as well as trophoblast cells, decidual cells, spiral artery smooth muscle cells, and endothelial cells (2, 32). There is evidence of cross-regulation between these immune cells, such that together they ensure correct uNK cell function (73, 74).

Our experiments imply that a primary cause of pregnancy loss in late gestation following Treg cell depletion in early pregnancy is placental insufficiency due to inadequate remodeling of decidual spiral arteries in early pregnancy. Spiral arteries from Treg cell–depleted mice had a smaller lumen and cross-sectional area and failed to exhibit the expected loss of α-SMA. This was accompanied by a trend toward increased relative wall thickness when Treg cells were depleted. Importantly, all these markers of spiral artery remodeling were normalized when mice were pretreated with WT Treg cells to prevent Treg cell deficiency.

A combination of immunohistochemical and RNA-sequencing data point to a role for Treg cells in modulating uNK cells. uNK cell subsets can be distinguished by their reactivity to DBA lectin, which detects GalNAc (75), a glycosylated structure acquired during functional maturation after conception (51, 52). DBA^+^ uNK cells are more abundant than DBA^–^ uNK cells in the decidua and exhibit pro-angiogenic activity. Treg cell depletion reduced DBA^+^ uNK cells in the decidua, and this was mitigated by Treg cell replacement. DBA^+^ uNK cells express higher levels of *Vegfa*, *Il22*, and *Pgf* compared with DBA^–^ uNK cells, consistent with a contribution to decidual angiogenesis, while DBA^–^ uNK cells express more *Ifng*, which also plays a role in remodeling of the decidual vasculature (35, 51).

We analyzed uNK cells utilizing a flow cytometry panel designed to detect trNK cells, cNK cells, and ILC1 cells, without regard to their DBA expression. trNK cells are resident within the decidua and express angiogenic factors, cNK cells are recruited from peripheral blood and are similar to splenic NK cells, while ILC1s arise from trNK cells residing in the nonpregnant uterus (76) but do not express the NK cell–specific transcription factor, EOMES (58, 77). Flow cytometry did not reveal any change in the relative proportion of the 3 uNK subsets in Treg cell–deficient mice. We therefore undertook RNA sequencing to assess the impact of Treg cell depletion on decidual gene expression. We demonstrate that when Treg cells are depleted, transcriptional changes indicating inappropriate NK cell and T cell activation arise, and mostly these were restored to varying degrees by Treg cell pretreatment.

Among the genes most strongly induced after Treg cell depletion were granzyme serine proteases that mediate NK cell– and T cell–mediated cytotoxicity. Granzymes can exert noncytotoxic enzymatic actions that cause tissue damage, remodel extracellular matrices, and induce pro-inflammatory cytokine release, so a range of consequences of elevated granzymes might contribute to the decidual changes seen after Treg cell depletion. While it is not possible from the data presented here to conclusively define the relative contribution of uNK cells versus T cells to these changes, for several reasons we consider that uNK cells are centrally involved. Previous studies show that T cells are a minor component of the decidual leukocyte population, whereas NK cells predominate (37, 78). That Treg cell depletion caused the prominent NK gene *Prf1* and NK cell transcription factors *Eomes*, *Id2*, and *Runx3* to be induced suggests an altered uNK cell transcriptional program. Our analysis did not indicate expansion of effector CD3^+^ or CD4^+^ T cells after Treg cell depletion. The decidua has specific mechanisms to exclude effector T cells by epigenetic silencing of chemokine genes *Cxcl9*, *Cxcl10*, and *Ccl5* (78), and no change in expression of these genes was seen after Treg cell depletion. Collectively, these data imply that uNK cells contribute to the altered decidual gene expression and the mechanisms underpinning impaired vascular remodeling. Further analysis will be needed to definitively understand how uNK cell functions are affected by Treg cells and how in turn this leads to altered vascular remodeling. It will also be important to evaluate whether specific effector T cell subsets such as CD8^+^ T cells and γδ T cells, which can express granzymes and potentially elicit fetal loss (79, 80), might also contribute to the decidual changes we observed after Treg cell depletion.

The RNA-sequencing data also point to altered decidual trophoblast invasion after Treg cell depletion. Several trophoblast genes were upregulated, including several associated with placental glycogen cells, while genes expressed by other extravillous trophoblast lineages were downregulated. This result is consistent with the histological data showing elevated abundance of glycogen cells, a specific subset of trophoblasts that store glycogen to serve late-gestation fetal growth in the phase just prior to parturition (81). Although there were no apparent effects of Treg cell depletion on placental weight in late gestation, the reduced fetal/placental weight ratio implies impaired placental efficiency. In several mouse models of complicated pregnancy, placental glycogen is not mobilized and instead is retained in the JZ (82–84). Retained placental glycogen has also been implicated in humans with preeclampsia and gestational diabetes (84).

There is extensive evidence of crosstalk between uNK cells and trophoblasts in the decidua. Trophoblasts influence the maturation of uNK precursor cells into mature uNK cells, and uNK cells both promote and constrain extravillous trophoblast migration and invasion of spiral arteries (85, 86). The reduced expression of trophoblast genes might imply excessive uNK cell constraint of trophoblast invasion after peri-implantation Treg cell depletion, but whether uNK cell cytotoxicity is involved will require further analysis. Although uNK cells express granzymes and *Prf1* even under normal circumstances, uNK cells do not exhibit killing of trophoblasts or fetal cells in the manner of peripheral blood NK cells (87), except under very specific circumstances, for example, in the event of viral (88) or bacterial infection (89). Given that in the current study, placental integrity was not overtly changed after Treg cell depletion, we consider uNK cell cytotoxic activity to be unlikely, and instead, noncytotoxic effects of uNK cell granzymes on trophoblast invasion and the decidual vasculature warrant investigation. Single-cell sequencing experiments will be informative in this regard.

Decidual gene expression analysis by qPCR revealed a potential role for cytokines TGF-β, IL-35, and IL-10 in communication between Treg cells and uNK cells. IL-35 is produced almost exclusively by Treg cells (90, 91) and has an established role in suppressing effector functions (92, 93), including in NK cells (68). IL-10 is an antiinflammatory cytokine produced by both immune and nonimmune cells, including Treg cells, uNK cells, macrophages, and trophoblasts (94). It is well established that IL-10 is critical for immune tolerance in pregnancy (95), and IL-10–null mutant mice exhibit elevated NK1.1^+^ decidual cell numbers and cytotoxic capacity (96). TGF-β can induce NK cell proliferation, upregulate NKp46 expression (97), and promote a more tolerogenic phenotype in NK cells (98). Reduced expression in each of these cytokines after Treg cell depletion presumably reflects their synthesis in Treg cells, particularly for IL-35, which is restricted to this cell lineage. Treg cell release of TGF-β, IL-10, and IL-35 are implicated in Treg cell–mediated protection of the vasculature in other tissues (99–103). Treg cells constrain vascular inflammation in atherosclerosis (20, 21), but whether Treg cell–NK cell communication affects vascular biology in other tissues is unknown. Such an interaction is possible given evidence of NK cell–Treg cell interactions in a range of settings (104–106).

Decidual gene expression analysis also revealed that *Vegfa* transcription was reduced in Treg cell–depleted mice. Given evidence that production of VEGFA by DBA^+^ uNK cells is important for angiogenesis and remodeling of the decidual vasculature (39), this finding is consistent with our histochemical data showing decreased DBA^+^ uNK cells. Our finding that IFN-γ remained unchanged after Treg depletion is consistent with Treg cells exerting a greater effect on DBA^+^ uNK cells producing VEGF than on DBA^–^ uNK cells secreting IFN-γ (39).

Along with decidual spiral artery remodeling, Treg cells are implicated in modifying the systemic vasculature and notably the uterine artery. After Treg cell depletion, we saw increased resistance to blood flow in the main uterine arteries in midgestation, and this was prevented with Treg cell replacement, verifying our earlier findings. Together with observations that Treg cell deficiency promotes generation of the vasoconstrictor endothelin-1 in uterine arteries (19), this demonstrates that Treg cells are necessary for normal function of the uterine artery in midgestation, as well as late gestation (17). The effects of Treg cell depletion on the systemic vasculature may be limited to the window during which Treg cells are deficient, as we found that uterine artery resistance was normalized after Treg cell populations recovered.

Collectively, our data on structural indicators of placental transport efficiency and compromised fetal growth in late pregnancy indicate a pathological state that is the consequence of early-gestation immune perturbation. This sequence of events is reminiscent of other pro-inflammatory perturbations in early pregnancy that manifest as impaired vascular remodeling, and altered placental development, followed by fetal loss and/or growth restriction in late gestation (30). That all the vascular defects, as well as fetal loss, could be rescued by transfer of WT Treg cells alleviates concern of off-target effects of DT and verifies that the consequences of DT treatment can be attributed to Treg cells. Although Treg cell replacement did not improve fetal weight, it did improve parameters of fetal growth, including crown-to-rump length, abdominal girth, and biparietal diameter; independent experiments in C57BL/6 mice showed no adverse impact on fetal growth of DT administration. This implies that the fetal weight deficit remaining after Treg replacement is due to incomplete recapitulation of the unperturbed immune state, as opposed to deleterious effects of DT. While these data clearly show an impact of Treg cells on the decidual vasculature and implicate a mediating role for uNK cells, we do not exclude the possibility that other effects of Treg cell depletion, mediated locally in the implantation site or systemically in other tissues, also contribute to fetal loss and growth restriction.

Impaired vascular remodeling of the maternal spiral arteries is the pathological antecedent of preeclampsia (13), resulting in poor uteroplacental perfusion later in pregnancy and often leading to fetal growth restriction. A possible role of Treg cells in facilitating the spiral artery–remodeling process has been speculated, but definitive evidence has been lacking (2, 107). Aberrations in uNK cells have been investigated as a possible contributing factor to impaired spiral artery remodeling in preeclampsia. There are conflicting reports on altered uNK cell numbers in preeclampsia (108–111), indicating the need for further research to resolve these conflicting observations.

**Figure 10:**
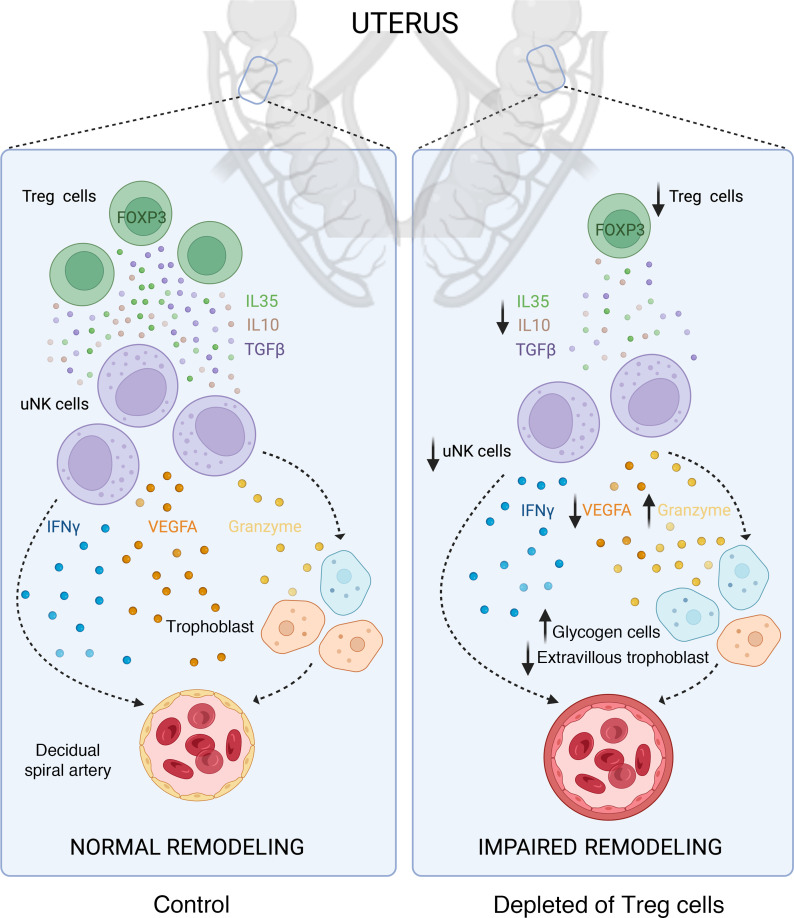
Schematic illustration of current working hypothesis on mechanisms of Treg cell–mediated regulation of decidual spiral artery remodeling. Treg cells modulate the decidual microenvironment to facilitate decidual spiral artery remodeling in early pregnancy in mice. When Treg cells are deficient, spiral artery remodeling is impaired. In turn this causes fetal growth restriction and late-gestation fetal loss that is exacerbated by increased resistance to blood flow in the uterine arteries. Treg cell support of spiral artery remodeling is likely to be mediated through Treg cell effects in a decidual network involving uNK cells and trophoblasts. Our data show reduced numbers of DBA^+^ uNK cells, and attenuation of genes associated with uNK cell function and extravillous trophoblast invasion. Since uNK cells are known to be essential for spiral artery remodeling through IFN-γ and VEGF production, and Treg cells produce cytokines TGF-β, IL-10, and IL-35 known to regulate uNK cell function, a direct effect of Treg cells on uNK cells is implicated. Altered extravillous trophoblast invasion and/or survival in the decidua may also be involved, since extravillous trophoblasts interact with uNK cells and contribute to spiral artery remodeling. See text for details. Created with BioRender.com.

In conclusion, this study provides evidence demonstrating that Treg cells are essential for remodeling of decidual spiral arteries in early pregnancy and identifies a candidate mechanism involving Treg cell regulation of uNK cells (Figure 10). Given the consistent finding of perturbed Treg cells in preeclampsia and related hypertensive pregnancy complications, these findings indicate that studies to determine effects of Treg cells on spiral artery remodeling in women are warranted and add to the imperative to consider Treg cells as a potential therapeutic target in at-risk women.

## Methods

### Sex as a biological variable.

Our study focused on pregnancy, and therefore, outcomes were focused on female mice. Male mice were used for mating purposes. Ongoing investigations are examining potential impacts on both male and female offspring.

### Animals.

*Foxp3*^DTR^ [B6.129(Cg)-*Foxp3^tm3(DTR/GFP)Ayr^*/J] mice were purchased from Jackson Laboratory, and C57BL/6 (WT) female and BALB/c male mice were purchased from Animal Resources Centre. Mice were housed and bred in a specific pathogen–free facility. Female mice (8–12 weeks) were housed with proven fertile males, and the presence of a copulatory plug was designated GD0.5. See Supplemental Methods for details.

### Treg cell depletion.

To induce Treg cell depletion, *Foxp3*^DTR^ mice were injected with DT from *Corynebacterium diphtheria* (MilliporeSigma) (37.5 ng/g, i.p.) on GD3.5 and GD5.5. Vehicle-treated (PBS) *Foxp3*^DTR^ mice were controls in all experiments. See Supplemental Methods for details.

### T cell isolation and adoptive transfer.

CD4^+^CD25^+^ Treg cells or CD4^+^CD25^–^ Tconv cells were isolated from the uDLNs; the mesenteric, inguinal, and brachial lymph nodes (LNs); and the spleen from pregnant WT C57BL/6 mice at GD10.5 to 13.5. Cells were adoptively transferred by i.v. injection into DT-treated *Foxp3*^DTR^ mice on GD3.5 and 5.5. See Supplemental Methods for details.

### Flow cytometry.

On GD6.5 and GD10.5, single-cell suspensions were prepared from uDLNs, mesenteric LNs, and pooled decidua from *Foxp3*^DTR^ mice. Cells were stained using fluorophore-conjugated antibodies to detect surface and intracellular markers (Supplemental Table 5) and a standardized gating strategy (Supplemental Figures 11 and 12). See Supplemental Methods for details.

### Ultrasound biomicroscopy.

Uterine artery function was assessed on GD9.5, and uterine and umbilical artery function were assessed at GD17.5, using an MX550D transducer (FUJIFILM VisualSonics) probe on an ultrasound biomicroscope (model Vevo 2100, FUJIFILM VisualSonics) in *Foxp3*^DTR^ mice. See Supplemental Methods for details.

### Histology, immunohistochemistry, and lectin staining.

Midsagittal sections from formalin-fixed, paraffin-embedded GD10.5 implantation sites (for decidual spiral artery analysis; Supplemental Figure 13) and GD18.5 placentas were stained with Masson’s trichrome. Smooth muscle cells were detected with α-SMA and uNK cells with biotinylated DBA-lectin. See Supplemental Methods for details.

### RNA sequencing and data analysis.

RNA from decidual tissues was extracted as described previously (12). Library preparation and sequencing on Illumina NovaSeq X at 80 million reads per sample was performed by the South Australian Genomics Centre. Sequencing reads were mapped to GRCm38 (mm10) mouse genome and quantified using STAR (112). Limma voom (113) was then used on TMM-normalized reads to identify genes that were differentially expressed (DEGs, FDR < 0.1) according to treatment group. Overrepresentation pathway analysis was performed with clusterProfiler (114). The Mouse Genomics Informatics resource (65) and mouse placenta single-cell sequencing data (66) were used to identify genes enriched in placental trophoblasts. See Supplemental Methods for details and data availability.

### Statistics.

Data were analyzed by unpaired 2-tailed *t* test or Mann-Whitney *U* test, or 1-way ANOVA or Kruskal-Wallis test, depending on normality of data distribution. Fetal outcome data were analyzed by mixed-model ANOVA with mother as subject and litter size as covariate. *P* < 0.05 was considered statistically significant. Analysis was performed using GraphPad Prism, SPSS, or R in the case of RNA-sequencing data. See Supplemental Methods for details.

### Study approval.

All experiments were approved by the University of Adelaide Animal Ethics Committee, ethics numbers M-2018-127, M-2017-024, and M-2020-107, in accordance with the *Australian Code of Practice for the Care and Use of Animals for Scientific Purposes* (8th edition, 2013). See Supplemental Methods for details.

### Data availability.

Values for all data points in graphs are reported in the Supporting Data Values XLS file. Complete R code used to analyze and visualize the RNA-sequencing data have been deposited at https://tranmanhha135.github.io/Treg_uNK/ Raw FASTQ files have been deposited in the National Center for Biotechnology Information’s Gene Expression Omnibus database (GSE267364).

## Author contributions

ASC and SAR designed the studies. SLH, HMG, HYC, HMT, LMM, EAKL, and ASC conducted experiments and analyzed data. SLH, ASC, HMT, and LMM prepared figures. KLF, CTR, and STD provided specific expertise and made substantial contributions to designing experiments and interpreting data. ASC and SAR wrote the manuscript. SLH, LMM, HMT, HYC, HMG, EAKL, ESG, SEO, CTR, KLF, STD, SAR, and ASC revised drafts and reviewed the manuscript.

## Supplementary Material

Supplemental data

Supporting data values

## Version 1 12/10/2024

In-Press Preview

## Version 2 01/23/2025

Electronic publication
