# Supplementary material for: Treg cells promote decidual vascular remodeling and modulate uterine NK cells in pregnant mice
Source: JCI Insight. 2024 Dec 10;10(2):e169836. doi: 10.1172/jci.insight.169836 (PMC11790030; doi:10.1172/jci.insight.169836)
Supplement: Supplemental data [file jciinsight-10-169836-s035.pdf]

Online Supplementary Information for:

Treg cells promote decidual vascular remodeling and modulate uterine NK cells in pregnant mice

Shanna L. Hosking[1], Lachlan M. Moldenhauer[1], Ha M. Tran[1], Hon Y. Chan[1], Holly M. Groome[1], Evangeline A. K. Lovell[1], Ella S. Green[1], Stephanie E. O'Hara[1], Claire T. Roberts[2], Karrie L. Foyle[1], Sandra T. Davidge[3], Sarah A. Robertson[1*], Alison S. Care[1*]

[1]Robinson Research Institute and School of Biomedicine, The University of Adelaide, Adelaide, SA, 5000, Australia

[2]Flinders Health and Medical Research Institute, Flinders University, Adelaide, SA 5042, Australia.

[3]Women and Children's Health Research Institute, Department of Obstetrics and Gynecology, University of Alberta, Edmonton, AB, T6G 2S2, Canada

*ASC and SAR are co-senior authors

Address correspondence to: Dr Alison S. Care, Robinson Research Institute and School of Biomedicine, The University of Adelaide, South Australia, 5000, Australia.
Ph: +61 883131504. Email: alison.care@adelaide.edu.au

This PDF file includes:

Supplementary Materials and Methods
Supplementary Table 1 to 5
Supplementary Figures 1 to 14

Supplementary Materials and Methods

Animals

Foxp3^{DTR} (Forkhead box P3-diphtheria toxin receptor; B6.129(Cg)-*Foxp3*$^{tm3(DTR/GFP)Ayr}$/J) (1) mice were purchased from Jackson Laboratories (Bar Harbour, Maine USA), and and bred at the Adelaide Medical School specific pathogen-free animal facility. The *Foxp3*^{DTR} is a knock-in transgenic mouse model that allows acute depletion of Treg cells after administration of diphtheria toxin (DT)(1). C57Bl/6 (WT) female and BALB/c male mice were purchased from Animal Resources Centre (Western Australia, Australia). Mice were provided food and water ad libitum and were housed under a 12-hour day-night cycle. Virgin wild-type or *Foxp3*^{DTR} female mice at 8-12 weeks of age were mated to proven fertile BALB/c male mice. Mating was confirmed by the presence of a vaginal plug between 0700-1000 h and was designated gestational day (GD) 0.5. Mated females were co-housed where possible, with a maximum of 2 females per cage. Mated mice were humanely killed via cervical dislocation on either GD6.5, 10.5 or 18.5. A subet of dams were allowed to deliver and birth outcomes were evaluated.

Treg cell depletion

To induce Treg cell depletion, *Foxp3*^{DTR} mice were injected with DT from *Corynebacterium diphtheria* (Sigma-Aldrich) i.p. on GD 3.5 and 5.5. Following a pilot dose titration experiment to evaluate doses of 10 to 50 ng/g, a dose of 37.5 ng/g (i.p), was chosen to achieve an adverse effect on fetal viability on GD10.5, but not complete pregnancy loss, as often occurred at the highest dose (2). Vehicle-treated (PBS) *Foxp3*^{DTR} mice were used as controls in all experiments. In some experiments wild-type C57BL/6 female mice were also treated with DT or vehicle control.

T cell isolation and adoptive transfer

For passive transfer experiments, $CD4^+CD25^+$ Treg cells or $CD4^+CD25^-$ Tconv cells were isolated from udLN, mesenteric LN, inguinal, and brachial lymph nodes and spleen from wild-type mice between GD 10.5 to 13.5, as previously described (3). Briefly, cells were purified using the EasySep™ Mouse $CD4^+CD25^+$ Regulatory T cell Isolation Kit II (STEMCELL Technologies, Vancouver) according to the manufacturer's instructions. Cells were >80% viable and were confirmed to be $CD4^+CD25^+$ cells (>75% purity, range 75-90%) or CD4+CD25- (>80% purity, range 81-96%) by flow cytometry using surface markers listed below in the flow cytometry section. Recipient *Foxp3*^{DTR} mice received 2-4 x 10^5 $CD4^+CD25^+$ Treg or $CD4^+CD25^-$ Tconv cells in PBS with 2% FCS intravenously via tail vein injection on the afternoon of GD2.5 and GD4.5. Control *Foxp3*^{DTR} mice were injected with vehicle (PBS with 2% FCS).

Pregnancy, fetal and birth outcomes

On GD10.5 and 18.5, the number of viable and resorbed implantation sites were recorded. On day 18.5, fetuses and placentae were weighed. Fetal crown-rump length, abdominal circumference and biparietal diameter were measured. One placenta from each uterine horn (total of 2 per dam) were fixed in 4% paraformaldehyde (Sigma-Aldrich) for further histological analysis. Implantation sites from GD10.5 were fixed, processed and cut as described previously (4). Placental tissue from GD18.5 were fixed, processed and cut as described previously (5). A subset of dams were allowed to give birth, and neonatal outcomes were recorded, including the number of pups born, pup viability, and pup bodyweight.

Fetal weight frequency distribution curves

Fetal weight, placental weight and ratio histograms were constructed as previously described (6, 7) and a non-linear regression performed (Gaussian distribution). The 10th percentile weights of the PBS-vehicle group were calculated as:
(-Z critical value x SD) + mean

Where Z critical value = 1.2816 (10th centile) and SD = standard deviation.

Flow cytometry

On GD6.5 and GD10.5, single-cell suspensions were prepared from spleen, uterine-draining para-aortic lymph nodes (udLN), mesenteric lymph nodes (MLN) and pooled decidua from *Foxp3*^{DTR} mice, as described previously (3). Decidua were digested with 1mg/mL collagenase VIII type, 200 U/mL Hyaluronidase, 1 mg/mL BSA dissolved in 5 mL HBSS, as described (8). Cells were washed in fluorescent-activated cell sorting (FACS) buffer (0.1% BSA, 0.05% sodium azide in PBS), filtered by 70 µm cell strainer and resuspended for cell counting.

1-2 x 10^6 cells of each sample were plated. Subsets of cells were stimulated as described previously (9). All samples were incubated with fixable viability stain (FVS) V-620 or R-780 and Fc receptor block (BD Biosciences). Cells were then washed, and a cocktail of monoclonal antibodies added (Supplemental Figure 12 and 13, and Supplemental Table 2). Cells were then fixed and permeabilized using the Intracellular Fixation and Permeabilization Buffer Set (eBioscience) according to the manufacturer's instructions and then labelled for the intracellular markers (Supplemental Table 2). Data were acquired on the BD LSRFortessa flow cytometer within 24 h of staining completion. Data were analysed using FlowJo analysis software version 7.6.1.

Ultrasound biomicroscopy

Uterine artery function was assessed on GD9.5 and uterine and umbilical artery function assessed at GD17.5, using an MX550D transducer probe on an ultrasound biomicroscope (model Vevo 2100, VisualSonics®, ON, Canada) in *Foxp3*^{DTR} mice. Doppler waveforms were obtained from uterine arteries near the lateral inferior margin of the utero-cervical junction, and umbilical arteries were taken from three fetuses per dam. Each waveform was analyzed in triplicate as described previously (2).

Histology, Immunohistochemistry and Lectin Staining

GD10.5 mid-sagittal implantation site sections and GD18.5 placental sections were stained with Masson's Trichrome, as described previously (10). To obtain mid-sagittal sections of GD10.5 implantation sites, serial sections were cut and those closest to the mid-sagittal plane were identified by visualization of the chorioallantoic attachment tissue linking the fetus and the placenta (Supplemental Figure 14)(4). To detect smooth muscle cells GD10.5 implantation sites were stained with α-SMA (0.5 µg/ml, Merck Millipore, Darmstadt, Germany) followed by secondary (3µg/ml, Vector Laboratories, Burlingame, CA, USA). To detect uNK cells, sections were stained with DBA-lectin (10 ug/mL, Vector Laboratories), followed by streptavidin-HRP and DAB chromogen (Dako) as described previously (11).

Slides were imaged using a Nanozoomer-XR Digital Slide Scanner (Hamamatsu Photonics, Hamamatsu, Japan). For analysis of decidual spiral arteries, SMA-positivity and uNK cell abundance, the inner two quarters of decidual regions from two implantation sites per dam were assessed with NDP.view2 software and Fiji (Image J; Version 2.0.0), as described previously (4). Proportion of positivity was considered as: (DBA-positive cells / (negative cells + positive cells) x 100).

Isolation of total RNA & mRNA expression analysis

Decidual tissues were homogenized and RNA extracted as described previously (12). Successful DNA removal was confirmed and RNA concentration was measured using a Nano-drop Spectrophotometer ND-1000 (Thermo Fisher Scientific), and RNA integrity was assessed by RNA Agilent Bioanalyzer (Agilent Technologies, Santa Clara, CA) to ensure all RNA samples had an RNA integrity number of >7. To measure mRNA expression, total DNase-treated RNA was reversed transcribed into cDNA from 800 ng RNA primed with 50 ng random hexamers using SuperscriptIV Reverse Transcription kit (Life

Technologies) according to the manufacturer's instruction. qPCR was performed using 20 ng cDNA and PCR primers:
Actb, 5' – TGTGATGGTGGGTATGGGTC, 3' – ACACGCAGCTCATTGTA; gDNA, 5' – GGCACTGACTGAGGT, 3' – GTCACAATCACAGAG; *Tgfb1*, 5'CTGAACCAAGGAGACGGAATACA, 3' – GGGCTGATCCCGTTGATTT; *Il10*, 5' – AGGCGCTGTCATCGATTTCT, 3' – TGGCCTTGTAGACACCTTGGT; *Ifng*, 5' – GCGTCATTGAATCACACCTG, 3' – TGAGCTCATTGAATGCTTGG; *Il12p35*, 5' – CTCCTGTGGGAGAAGCAGAC, 3' – CAGATAGCCCATCACTTTCT; *Ebi3*, 5' – GCTCCCCTGGTTACACTGAA, 3' – ACGGGATACCGAGAAGCAT; *Ncr1*, 5' – TGGCTCTTACAACGACTATGC, 3' – AGAAGAAGTAGGGTCGGTAGGTG; *Foxp3*, 5' – GGCAGAGGACACTCAATGAAATCTA, 3' – TGGCGGATGGCATTCTTC; and *Vegfa*, 5' – CATCTTCAAGCCGTCCTGTGT, 3' – CGCATGATCTGCATGGTGAT, as described previously (13). The delta C(t) method was used to calculate mRNA abundance normalized to *Actb* expression (14).

RNA sequencing and analysis

Sequencing libraries were prepared from high-integrity RNA (RIN>7) using the Illumina TruSeq stranded protocol and sequenced on the Illumina NovaSeq X platform at a depth of ~80 million reads per sample. Sequencing FASTQ files were processed with a standard RNA-seq pipeline. Sequence read quality was accessed by *FastQC* (v0.12.1, (15), trimmed with *cutadapt* (v.1.18,(16)) and aligned to GRCm38 (mm10) mouse genome and reads quantified using STAR (v2.7.10b_alpha_230301, (17)). Low-expressed genes (<2 counts per million (CPM) in at least 4 samples) were removed and normalized using the trimmed mean of M value (TMM) method through the *edgeR* (v.3.42.4, (18)) package. Differential gene expression analysis was performed using *limma-voom* (19) where significant differentially expressed genes defined as those with FDR-adjusted P value (FDR) <0.1. To investigate gene pathways and upstream regulators influenced during the peri-implantation period of pregnancy, differentially expressed genes were analysed using Ingenuity Pathway Analysis (IPA) version 65367011 Build ing_diamond (Ingenuity Systems, Redwood City, CA). Overrepresentation analysis was performed with *clusterProfiler* (v4.8.3, (20) using Gene Ontology (GO, (21), Kyoto Encyclopaedia of Genes and Genomes (KEGG, (22), Reactome (23), and Ingenuity Pathway Analysis (IPA, QIAGEN) databases.

Data availability

Complete R code used to analyze and visualize the RNA-sequencing data have been deposited at https://tranmanhha135.github.io/Treg_uNK/. Raw fastq files have been deposited in the National Center for Biotechnology Information's Gene Expression Omnibus database (GSE267364).

Statistics

All data were tested for normality of distribution using the Shapiro-Wilk normality test. Data were analysed by unpaired t-test or Mann-Whitney U when comparing two groups or with a one-way ANOVA or Kruskal-Wallis test depending on normality. All statistical tests were 2-sided. Fetal outcome data were analysed by mixed model ANOVA analysis with mother as the subject and litter size as a covariate. All statistical analysis was conducted using GraphPad Prism 9.4.0 for MacOS, software (GraphPad Software, La Jolla, CA) or IBM SPSS Statistics 28.0.1.0. Differences between groups were considered significant when P<0.05. For one-way ANOVA analyses, the following pre-selected group comparisons were conducted with multiple comparisons: vehicle vs. DT, vehicle vs. Treg, vehicle vs. Tconv, and DT vs. Treg, corrected with Šídák test. Categorical data were analysed using chi-square tests.

Study Approval

All experiments performed as part of this project were approved by the University of Adelaide Animal Ethics Committee, ethics numbers M-2018-127, M-2017-024 and M-2020-107 in accordance with the Australian Code of Practice for the Care and Use of Animals for Scientific Purposes (8th edition, 2013). All breeding of mice involved in this project were conducted under the ethics approval number M-2016-009 and M-2019-087.

Supplemental Table 1: Effect of Treg cell depletion and replacement on parameters of uterine and umbilical artery function as measured by Doppler ultrasound on GD17.5.

	PBS+veh	DT+veh	DT+Treg	DT+Tconv
UT PSV	361.3 ± 45.4	351.8 ± 42.8	367.2 ± 25.8	423.7 ± 39.6
UT EDV	132.0 ± 28.0	130.8 ± 26.0	135.4 ± 18.3	180.8 ± 22.7
UT VTI	242.0 ± 35.7	236.9 ± 34.3	252.8 ± 21.8	301.0 ± 28.9
UT PI	1.04 ± 0.09	1.04 ± 0.09	0.98 ± 0.09	0.82 ± 0.08
UT RI	0.66 ± 0.04	0.66 ± 0.04	0.64 ± 0.04	0.57 ± 0.03
UM PSV	156.7 ± 9.9	164.7 ± 10.8	147.1 ± 7.7	166.5 ± 15.3
UM EDV	10.5 ± 1.3	9.2 ± 1.5	9.7 ± 1.1	12.0 ± 1.2
UM VTI	81.5 ± 5.4	87.0 ± 7.0	77.9 ± 4.5	89.8 ± 8.8
UM PI	1.80 ± 0.03	1.82 ± 0.03	1.78 ± 0.03	1.73 ± 0.03
UM RI	0.93 ± 0.01	0.95 ± 0.01	0.94 ± 0.01	0.93 ± 0.01

UT, uterine artery; PSV, peak systolic velocity; EDV, end diastolic velocity; VTI, velocity time interval; PI, pulsatility index; RI, resistance index; UM, umbilical artery. N=12-15 dams per group.
Data are presented as mean±SEM. Data points are average values for individual dams. Analysis was by one-way ANOVA or a Kruskal-Wallis test, with two-tailed posthoc t-test.

Supplemental Table 2: RNA-sequencing data metrics for decidua on day 10.5 pc following peri-implantation Treg depletion (DT), vehicle treatment (veh) or early pregnancy Treg replacement after peri-conception Treg depletion (DT+Treg).

Sample	Library size
DT1	436870
DT2	515936
DT3	695441
DT4	590735
DT5	846531
veh1	610235
veh2	580250
veh3	584390
veh4	659764
veh5	698543
veh6	737791
DT+Treg1	710762
DT+Treg2	685291
DT+Treg3	702458
DT+Treg4	686864
DT+Treg5	727353

Supplemental Table 3: Top 50 genes upregulated identified in DT vs PBS and their expression in DT+Treg vs DT.

Gene symbol	**DT vs veh**		**DT+Treg vs DT**	
	logFC	p.adj	logFC	p.adj
2310020H05Rik	2.44	0.032	-1.208	0.314
Tgtp1	2.34	0.098	-0.256	0.896
Vcam1	2.21	0.025	-0.942	0.347
Gbp8	2.14	0.019	-1.038	0.231
Dnah8	2.12	0.062	-2.213	0.078
Ddx60	2.09	0.077	-0.909	0.481
Mir6397	2.07	0.062	-0.570	0.700
Tgtp2	2.07	0.019	-1.005	0.253
Zfp788	2.02	0.077	-1.016	0.414
Med18	1.98	0.091	-0.857	0.515
Serpina3f	1.95	0.086	-1.298	0.301
Nxnl2	1.93	0.065	-0.681	0.577
Inhba	1.86	0.070	-0.712	0.517
Gm12185	1.86	0.093	-2.194	0.068
Aplp1	1.84	0.086	-0.611	0.613
Apol9a	1.80	0.050	-1.006	0.270
H2-T10	1.71	0.054	-0.472	0.637
Glt8d2	1.60	0.091	-0.579	0.600
Ctsg	1.57	0.047	-1.011	0.206
Ceacam10	1.45	0.038	-0.440	0.573
Havcr2	1.44	0.054	-1.000	0.205
Iigp1	1.37	0.002	-0.765	0.055
Gbp6	1.37	0.062	-1.498	0.063
Apol10b	1.33	0.077	-0.326	0.720
Zbp1	1.28	0.015	-0.640	0.225
Gzmf	1.28	0.007	-1.124	0.021
Ctsk	1.23	0.044	-1.083	0.124
Rasal3	1.22	0.019	-0.507	0.351
Nlrc5	1.22	0.012	-1.202	0.015
Med7	1.21	0.032	-0.460	0.480

Olr1	1.18	0.005	-0.589	0.192
Map7d2	1.17	0.028	-0.886	0.129
Lax1	1.14	0.077	-0.925	0.182
Atp1a3	1.14	0.050	-1.046	0.101
Gbp4	1.12	0.020	-1.298	0.015
Ifi44	1.12	0.077	-0.440	0.545
C2	1.10	0.005	-0.772	0.054
AW112010	1.08	0.012	-0.539	0.220
Gzmg	1.08	0.039	-1.239	0.035
Kng2	1.07	0.019	-0.543	0.279
H2-Q4	1.07	0.000	-0.387	0.179
Gzme	1.05	0.034	-0.983	0.072
Ccr2	1.04	0.034	-0.389	0.506
Zap70	1.04	0.018	-0.876	0.054
Mir6363	1.03	0.015	-0.154	0.792
Ifi47	1.01	0.093	-0.302	0.688
Gm21083	1.01	0.019	-0.542	0.231
Tnfrsf9	1.01	0.012	-0.773	0.062
Card11	0.99	0.038	-0.474	0.377
Tap1	0.98	0.001	-0.312	0.295

Supplemental Table 4: Top 50 genes downregulated identified in DT vs PBS and their expression in Treg vs DT.

Gene symbol	DT vs veh		DT+Treg vs DT	
	logFC	p.adj	logFC	p.adj
Aqp5	-4.018	0.005	-0.609	0.856
1700003G13Rik	-3.419	0.013	0.773	0.760
Casr	-3.411	0.007	-0.304	0.921
Nkx6-3	-3.410	0.019	0.156	0.969
Gm14023	-3.329	0.014	0.601	0.827
Klk1	-3.240	0.013	-1.539	0.623
Cyp26a1	-3.225	0.012	0.310	0.917
Car4	-3.196	0.019	-1.370	0.680
Slc46a2	-3.111	0.029	0.507	0.866
Ces2b	-3.027	0.005	-0.720	0.739
Prss28	-2.988	0.021	-0.230	0.947
5330417C22Rik	-2.941	0.032	-1.127	0.742
Sel1l3	-2.815	0.017	-0.505	0.848
Prss29	-2.796	0.045	-1.627	0.609
Padi4	-2.766	0.020	0.647	0.776
Slc6a14	-2.728	0.034	-0.061	0.987
Trpv5	-2.713	0.029	-0.601	0.832
Fzd10	-2.675	0.027	0.013	0.996
Tmem213	-2.671	0.033	-0.110	0.976
Cyp26c1	-2.657	0.019	-0.416	0.881
Il31ra	-2.648	0.019	-0.168	0.953
Vstm5	-2.646	0.038	1.029	0.623
Ckmt1	-2.620	0.024	-0.480	0.857
Mab21l3	-2.613	0.062	-0.110	0.978
Elovl2	-2.583	0.062	-0.914	0.754
Gm5294	-2.567	0.024	-0.561	0.817
1700028J19Rik	-2.564	0.062	-0.947	0.714
Pcsk6	-2.557	0.024	0.028	0.992
Fam13a	-2.553	0.026	-0.802	0.742
Dio2	-2.550	0.030	0.188	0.949
Tmprss4	-2.529	0.046	-1.061	0.690
Adamts7	-2.486	0.009	1.027	0.448

Pinlyp	-2.478	0.077	-1.062	0.742
C2cd4a	-2.468	0.019	-0.102	0.972
Lrat	-2.443	0.039	-0.738	0.777
Slc13a2os	-2.433	0.032	-0.403	0.885
Barx2	-2.416	0.071	-0.101	0.977
Grhl2	-2.409	0.045	-1.453	0.514
Fcamr	-2.403	0.100	-0.386	0.911
Hnf1b	-2.390	0.041	-0.281	0.925
Sgms2	-2.373	0.003	-0.259	0.879
Serpina1e	-2.372	0.065	-0.778	0.784
Ch25h	-2.372	0.018	0.095	0.971
Aqp8	-2.367	0.027	-1.220	0.564
Slc5a9	-2.362	0.063	-0.470	0.864
Sv2b	-2.358	0.082	-0.260	0.934
Atp12a	-2.323	0.028	0.388	0.856
Rgs7bp	-2.311	0.062	1.526	0.339
Dlx5	-2.301	0.049	-0.764	0.739
Gabrp	-2.296	0.062	-0.415	0.889

Supplemental Table 5: Monoclonal antibodies used in flow cytometry experiments

Antigen	Conjugate	Clone	Source	CAT#	Concentration (µg/mL)
Surface labelling:					
CD4	BUV496	GK1.5	BD	612952	0.67
CD4	BUV395	GK1.5	BD	563790	0.67
CD3	FITC	17A2	BD	555274	10
CD45	BUV395	30-F11	BD	564279	1.0
CD25	PE-Cy7	PC61	BD	552880	2.0
CD305 (Nrp1)	V421	3E12	Biolegend	145209	0.33
CD19	BUV737	1D3	BD	612782	0.67
NK1.1	BV605	PK136	BD	563220	1.67
F4/80	PE	T45-2342	BD	565410	1.33
CD335 (NKp46)	PerCP-Cy5.5	29A1.4	BD	560800	2.0
CD11b	BV786	M1/70	BD	740861	1.67
CD49a	BV421	Ha31/8	BD	740046	2.0
Intracellular labelling:					
EOMES	AF647	X4-83	BD	567168	0.2
FOXP3	APC	FJK-16S	Invitrogen	17-5773-82	0.2
CTLA4	PE	UC10-4F10-11	BD	553720	0.2
Ki67	R718	B56	BD	566963	0.5
Ki67	FITC	SolA15	Invitrogen	11-5698-82	0.5
IFNɣ	PE-CF594	XMG1.2	BD	562333	0.2

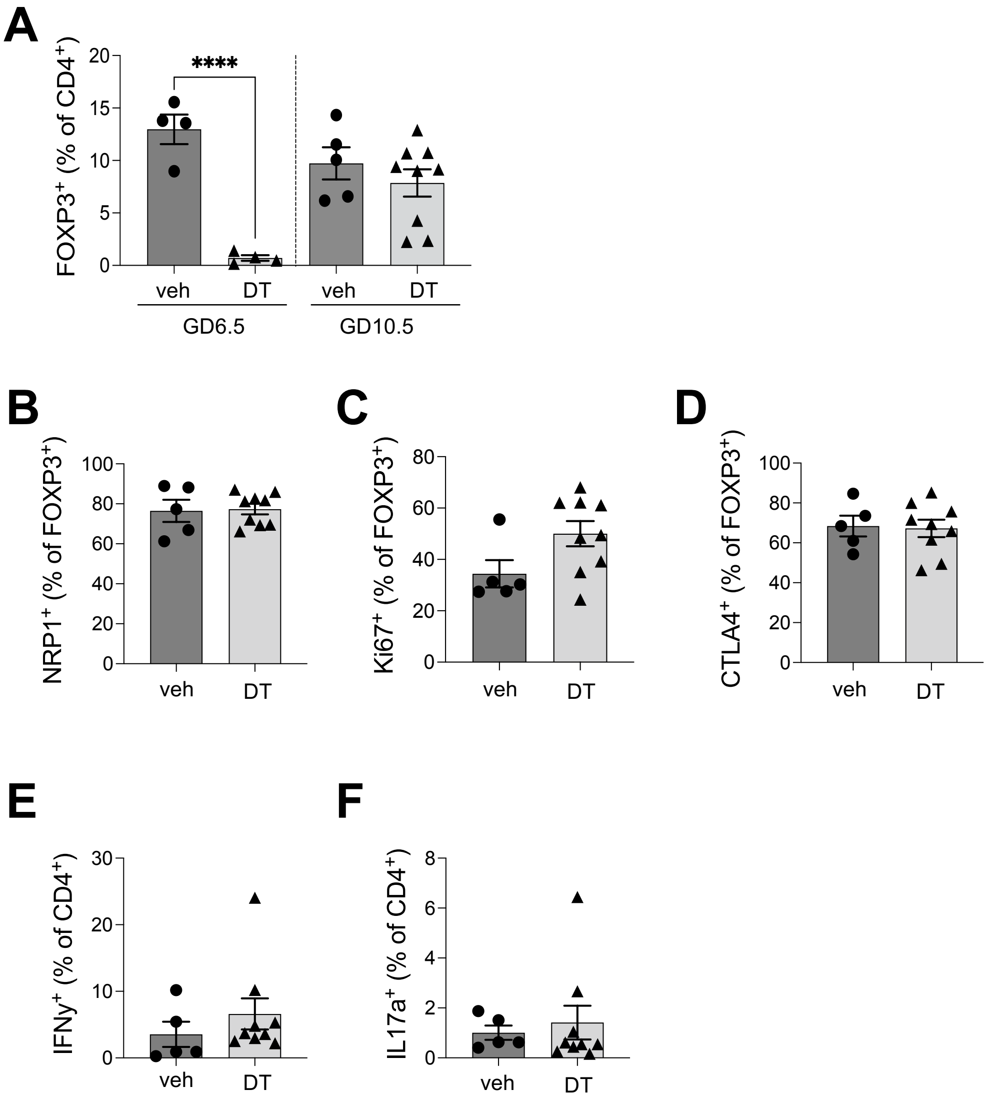

Supplemental Figure 1: Effect of DT administration to *Foxp3*^{DTR} mice in the peri-implantation period on spleen Treg cell proportion and phenotype in mid-gestation. *Foxp3*^{DTR} mice were administered PBS (veh) or DT i.p. on GD3.5 and GD5.5. Spleens were recovered on GD6.5 or GD10.5 and the proportion and phenotype of $CD4^+FOXP3^+$ Treg cells were evaluated using flow cytometry. (**A**) The proportion of $CD4^+FOXP3^+$ Treg cells in spleen at GD6.5 and GD10.5. Detailed analysis of Treg cells at GD10.5 shows the proportion of Treg cells expressing NRP1 indicating thymic origin (**B**), proliferation marker Ki67 (**C**), and marker of suppressive competence CTLA4 (**D**). $IFN\gamma^+CD4^+$ (Th1 cells; **E**) and $IL17a^+CD4^+$ (Th17 cells; **F**) were measured. N=4-9 mice per group. Data are presented as mean±SEM. Data points are values from individual dams. Analysis was by two-tailed t-test or Mann Whitney U test depending on normality of data distribution for (B-F). Data in (A) were analysed using a 1-way ANOVA comparing samples within the same gestational day. ****P<0.0001.

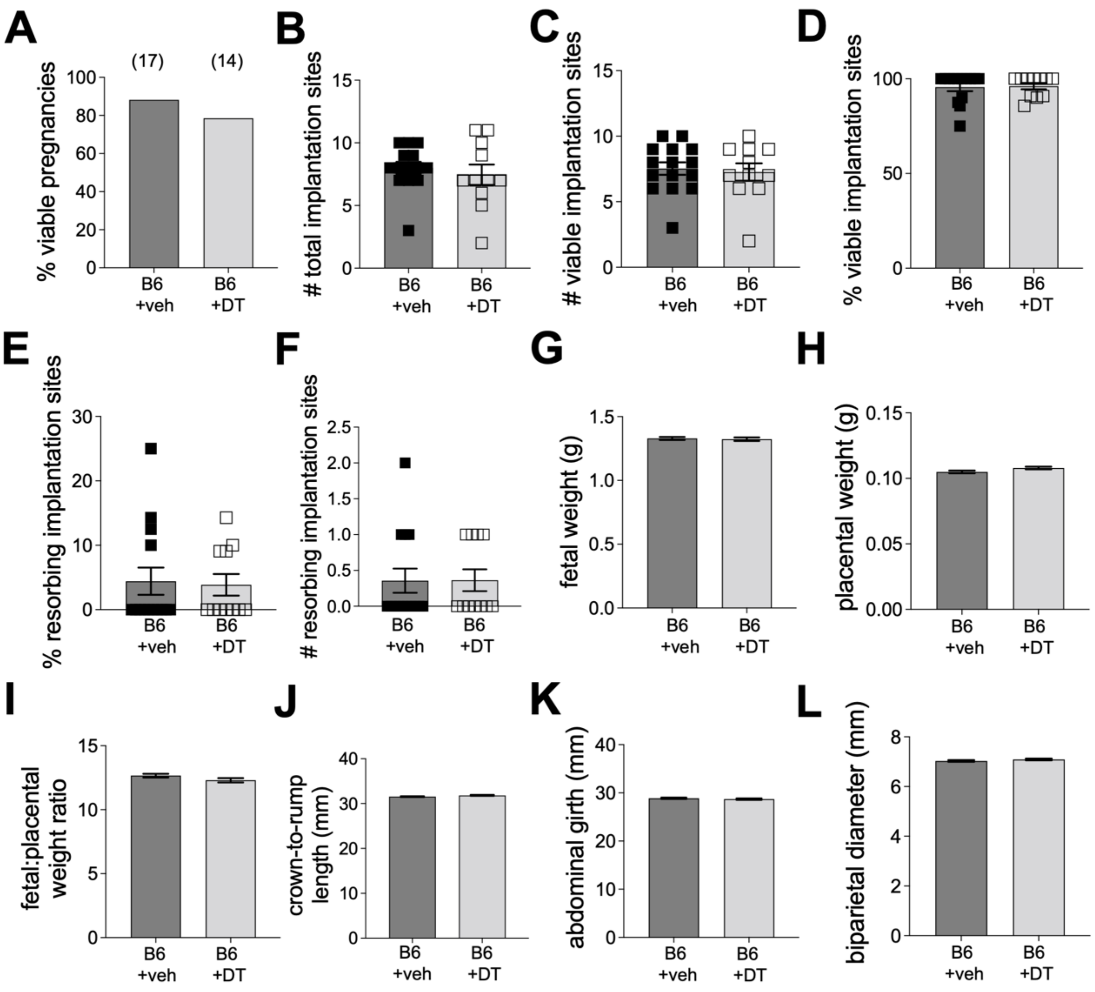

Supplemental Figure 2: DT administration to C57Bl6/J mice in the peri-implantation period does not alter pregnancy outcome or fetal growth. Pregnant C57Bl6/J female mice were administered PBS (veh) or DT i.p. on GD3.5 and GD5.5, then analyzed on GD18.5 for pregnancy and fetal outcomes. (**A**) Proportion of dams carrying viable pregnancies; (**B**) total implantation site number per dam; (**C**) number of implantation sites considered viable; (**D**) proportion of all implantation sites considered viable per dam. (**E**) Proportion, and (**F**) number of resorbing implantation sites per dam. (**G**) fetal weight; (**H**) placental weight; (**I**) fetal to placental weight ratio; (**J**) crown to rump length; (**K**) abdominal girth; and (**L)** biparietal diameter. Numbers of mice in each group are shown in parentheses. Data are presented as mean±SEM and were analysed using chi-squared test (A), or two-tailed t-test (B-F). For G-L, data are presented as estimated marginal mean±SEM and were analysed using a mixed model analysis with litter size as covariate.

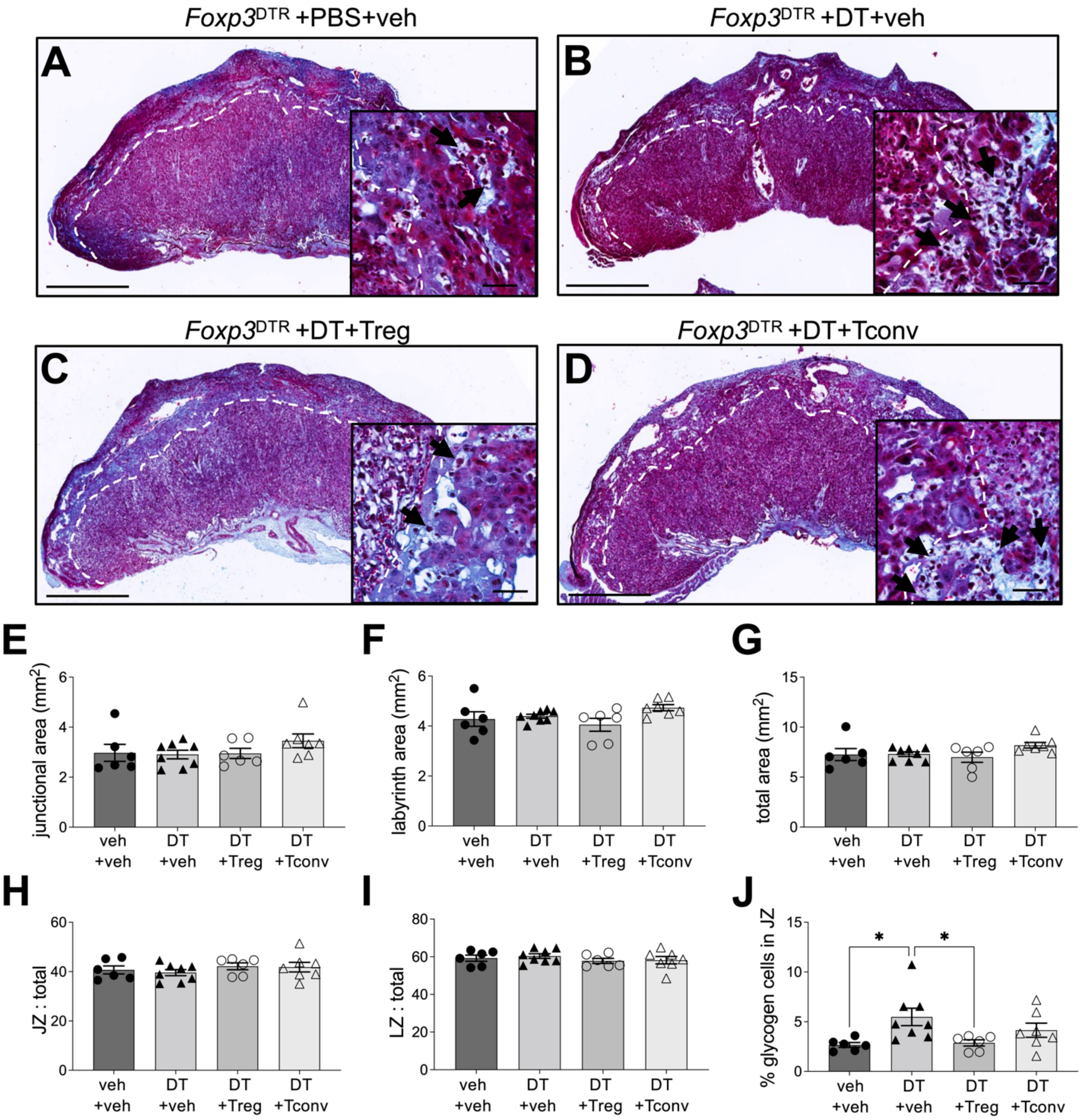

Supplemental Figure 3: Treg depletion increases placental glycogen cell retention in late gestation. Pregnant *Foxp3*^{DTR} mice were administered PBS (veh) or DT i.p. on GD3.5 and GD5.5. Some mice also received wild-type Treg cells or Tconv cells on GD2.5 and GD4.5. Tissue was collected on GD18.5. (**A** to **D**) representative images of placental mid-sagittal sections, with black arrows within the insets showing junctional zone glycogen cells. (**E**) junctional zone area, (**F**) labyrinth zone area, and (**G**) total placental area were measured. From this, (**H**) junctional zone to total area ratio, (**I**) labyrinth zone area to total area ratio were calculated. (**J**) the proportion of glycogen cells to total junctional zone area were measured. N=2 placentas per dam, 6-8 dams per group. Data are presented as mean±SEM. Data points are average values for individual dams. Significance was tested with a one-way ANOVA with two-tailed posthoc t-test. *P<0.05. Scale bars: outset = 1 mm, inset = 50 μm.

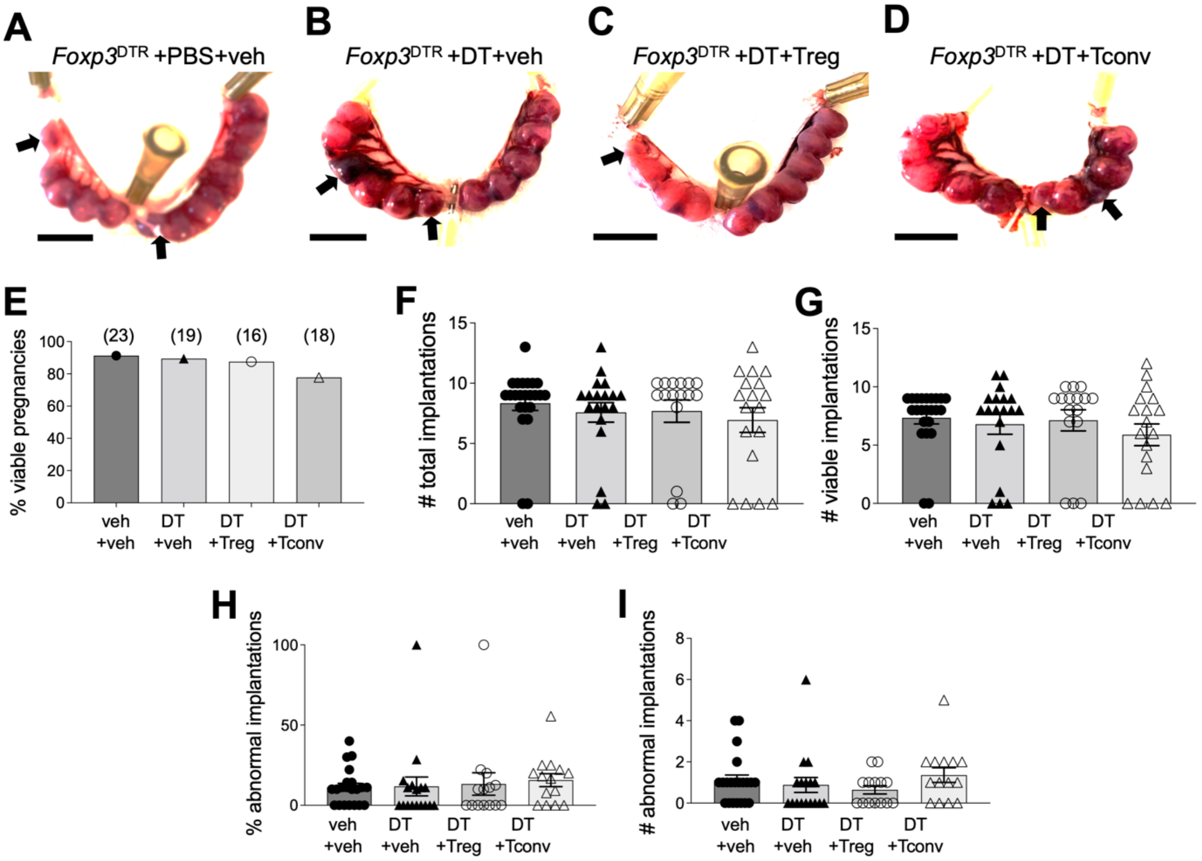

Supplemental Figure 4: Treg cell depletion in the peri-implantation period does not impact number of implantation sites in mid-gestation. Pregnant *Foxp3*^{DTR} mice were administered PBS (veh) or DT i.p. on GD3.5 and GD5.5, then tissues were collected on GD10.5. Some mice also received wild-type Treg cells or Tconv cells on GD2.5 and GD4.5 (**A** to **D**) representative images of uteri on GD10.5. Black arrows indicate abnormal implantation sites. (**E**) Of all mice with a detected copulatory plug, the proportion of those mice carrying a viable pregnancy (one-or-more viable implantation site) at mid-gestation was calculated. (**F**) The absolute number of implantation sites. (**G**) the number of viable implantation sites. (**H**) the proportion of abnormal implantation sites. (**I**) the number of abnormal implantation sites per dam was calculated. Number of dams in each group is shown in parentheses. Data are presented as mean±SEM. Data points are average values for individual dams. Analysis was by chi-squared analysis or one-way ANOVA with two-tailed posthoc t-test. Scale bar = 1 cm.

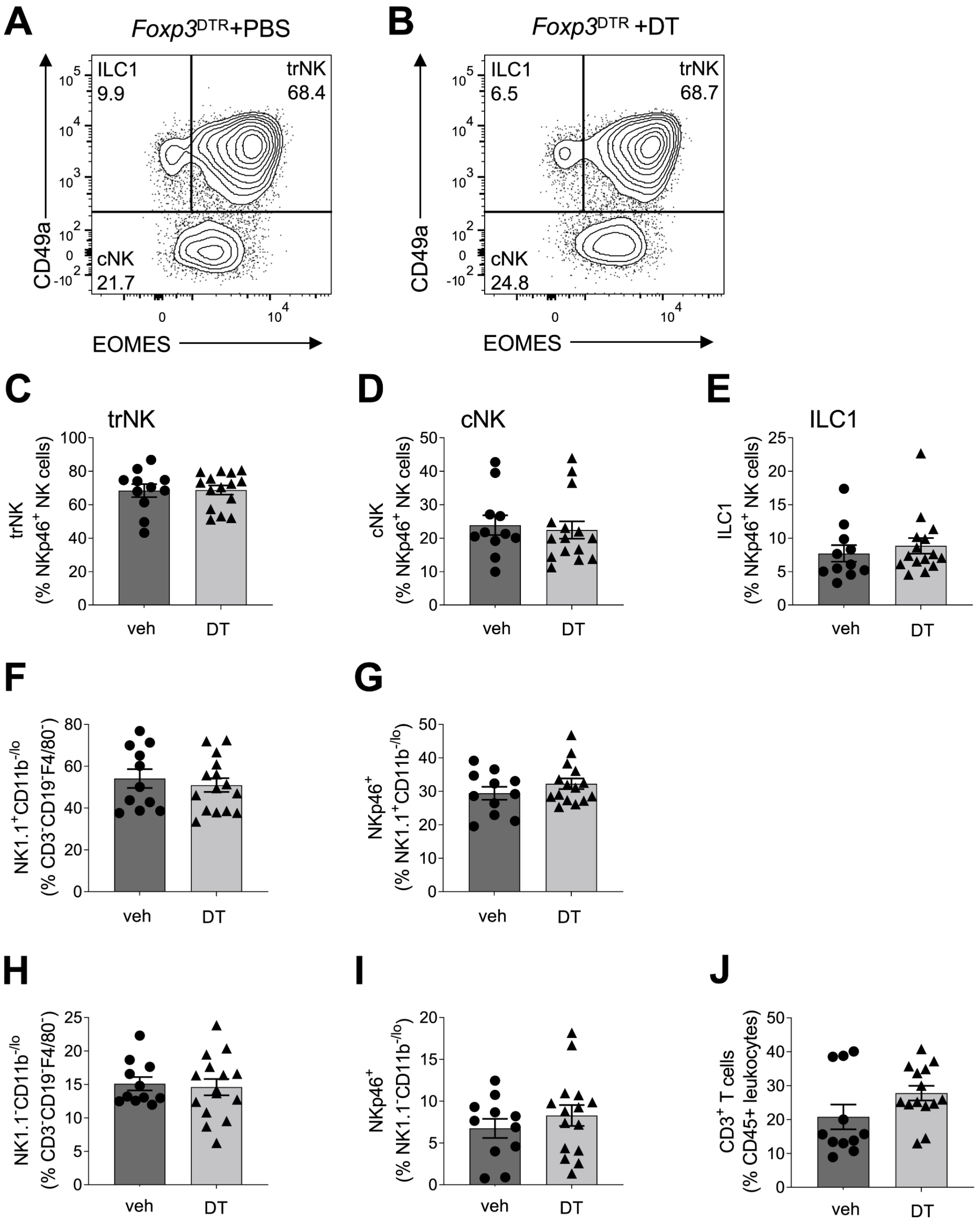

Supplemental Figure 5: Flow cytometry analysis shows the proportion of decidual uNK cell subpopulations at mid-gestation does not change in response to Treg cell depletion. Pregnant *Foxp3*^{DTR} mice were administered PBS (veh) or DT i.p. on GD3.5 and GD5.5. Decidua was collected on GD10.5 and analysed by flow cytometry. The expression of CD49a and EOMES within NK1.1^{+}CD11b$^{lo/-}$NKp46^{+} uNK cells was assessed, with representative plots shown (**A**,**B**), to calculate the proportion of CD49a^{+}EOMES^{+} tissue resident NK cells (trNK, **C**), CD49a^{-} conventional NK cells (cNK, **D**), and CD49a^{+}EOMES^{-} type 1 innate lymphoid cells (ILC1, **E**). The proportion of NK1.1^{+}CD11b$^{-/lo}$ uNK cells within the viable CD45^{+}CD3^{-}CD19^{-}F4/80^{-} (**F**), and the percentage of NKp46^{+} uNK cells (**G**), were measured. The percentage of NK1.1^{-}CD11b$^{-/lo}$ cells within viable CD45^{+}CD3^{-}CD19^{-}F4/80^{-} cells (**C**), and the proportion of these cells expressing NKp46 (**D**) were measured. The number of CD3^{+} T cells was also determined (**J**). N=11-15 dams per group. Data are presented as mean±SEM. Data points are average values for individual dams. Analysis was by two-tailed t-test.

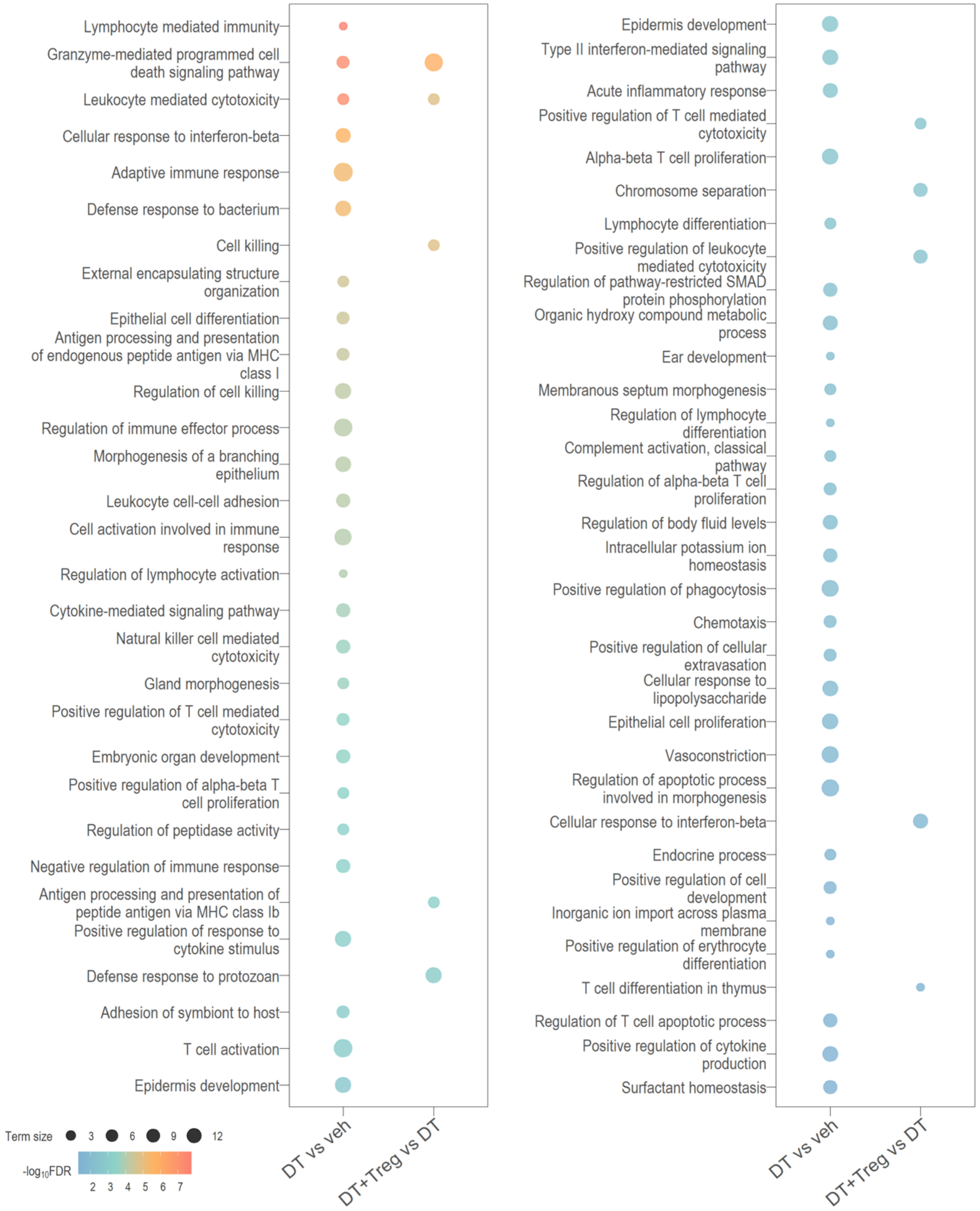

Supplemental Figure 6: Enriched Gene Ontology (GO) biological processes parent terms. Pregnant *Foxp3*^{DTR} mice were given PBS (veh; N=6) or DT (N=5) i.p. on GD3.5 and GD5.5, and decidual tissue was collected on GD10.5. Some mice (N=5) also received Treg cells isolated from wild-type pregnant mice on GD2.5 and GD4.5. Significant DEGs (FDR <0.1) were analysed for enriched GO terms followed by semantic similarity analysis to reduce similar terms into parent terms. Symbol size is proportional to the number of significant GO terms within each parent term and colour indicates -log$_{10}$FDR.

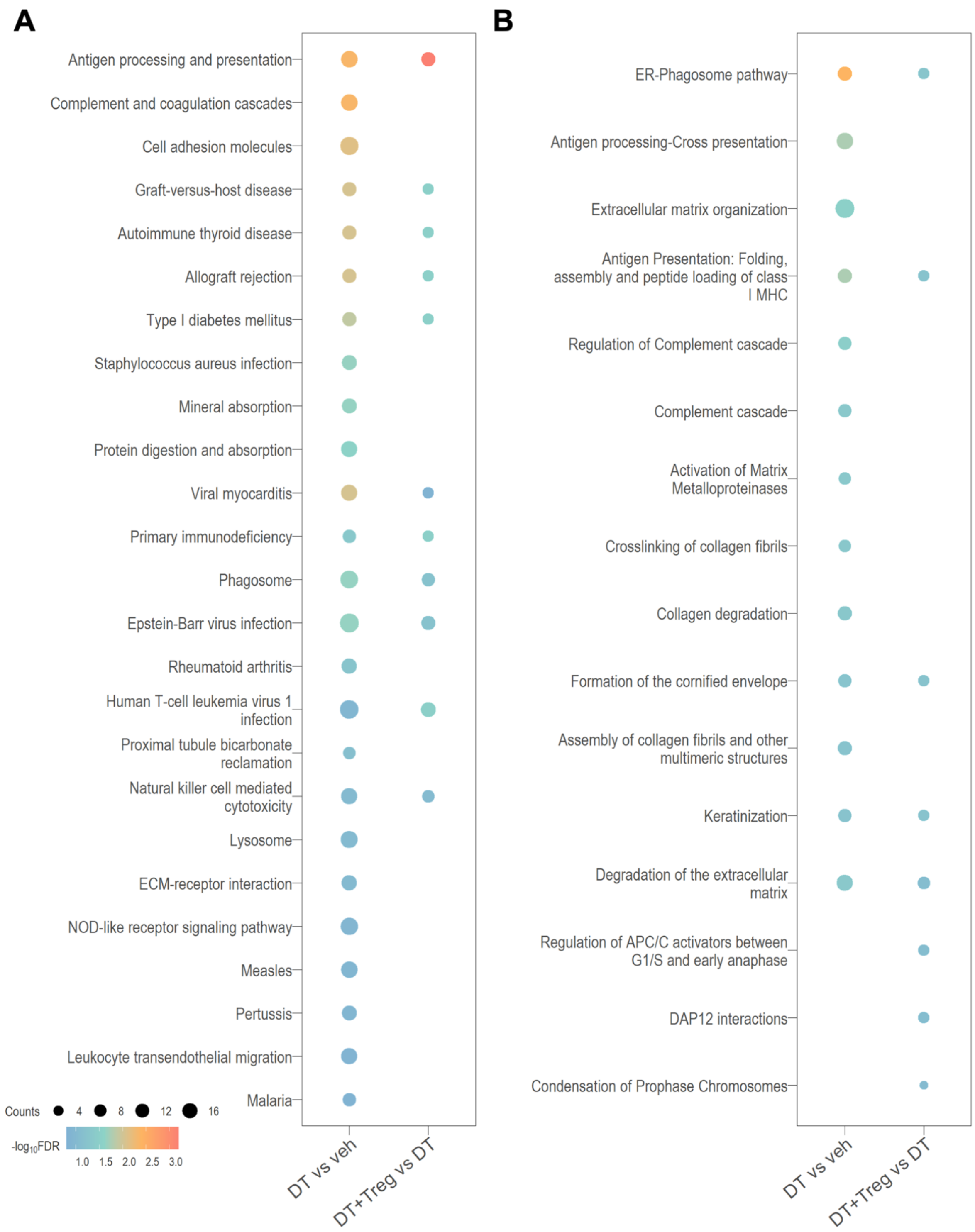

Supplemental Figure 7: Overrepresentation analysis of Kyoto Encyclopedia of Genes and Genomes (KEGG) and Reactome pathways. Pregnant *Foxp3*^{DTR} mice were given PBS (veh; N=6) or DT (N=5) i.p. on GD3.5 and GD5.5, and tissues collected on GD10.5. Some mice (N=5) also received Treg cells isolated from wild-type pregnant mice on GD2.5 and GD4.5. Significant DEGs (FDR <0.1) were analysed for enriched (A) KEGG and (B) Reactome pathways. Symbol size is proportional to the number of significant DEGs in each pathway and colour indicates -log$_{10}$FDR.

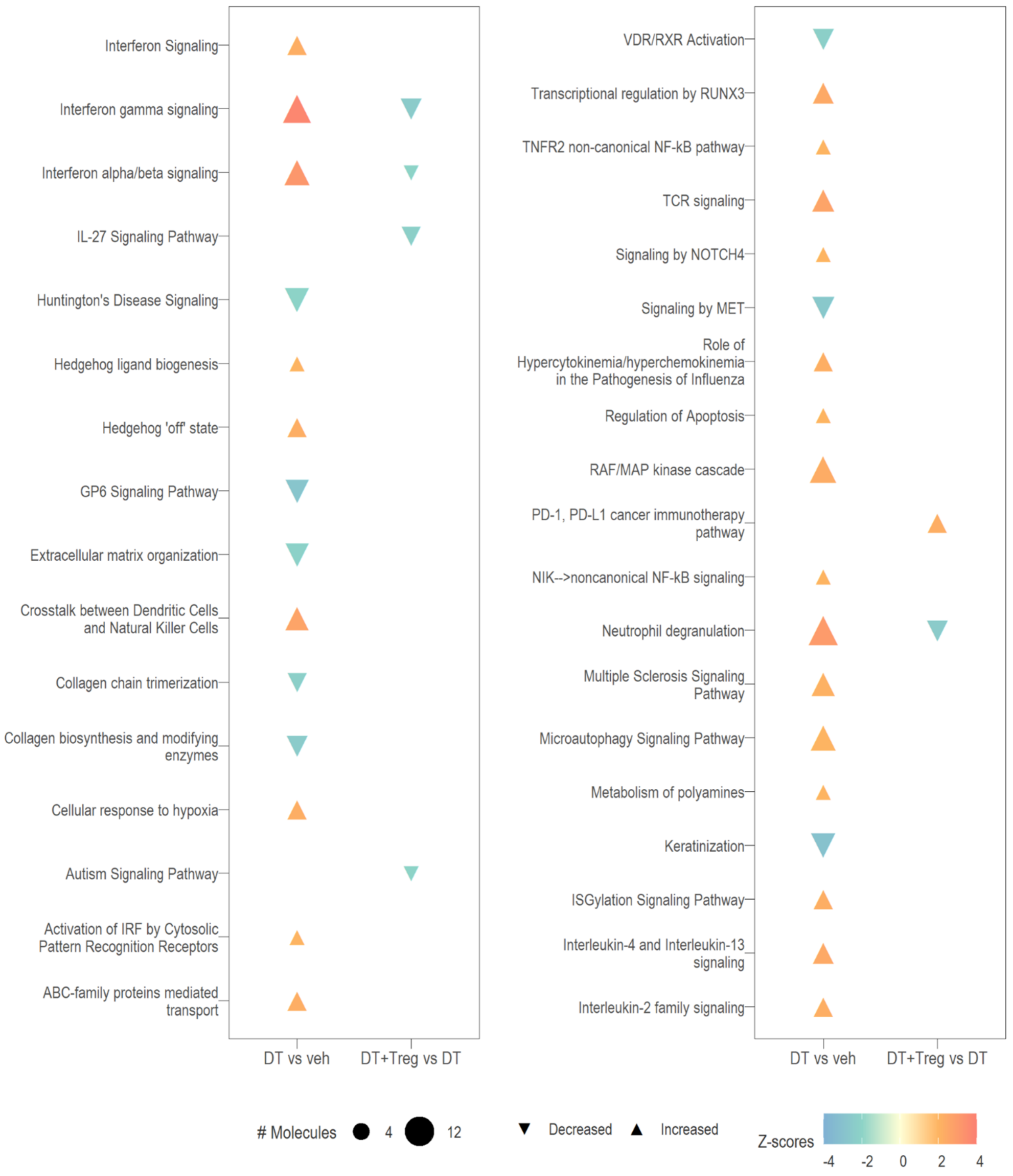

Supplemental Figure 8: Ingenuity Pathway Analysis (IPA) canonical pathways activity. Pregnant *Foxp3*^{DTR} mice were administered PBS (veh; N=6) or DT (N=5) i.p. on GD3.5 and GD5.5, and tissues collected on GD10.5. Some mice (N=5) also received Treg cells isolated from wild-type pregnant mice on GD2.5 and GD4.5. Significant DEGs (FDR < 0.1) were analysed for enriched IPA pathways. All pathways are significant (*P* <0.05) with z-score indicating the predicted activation state. Symbol size is proportional to the number of significant DEGs in each pathway.

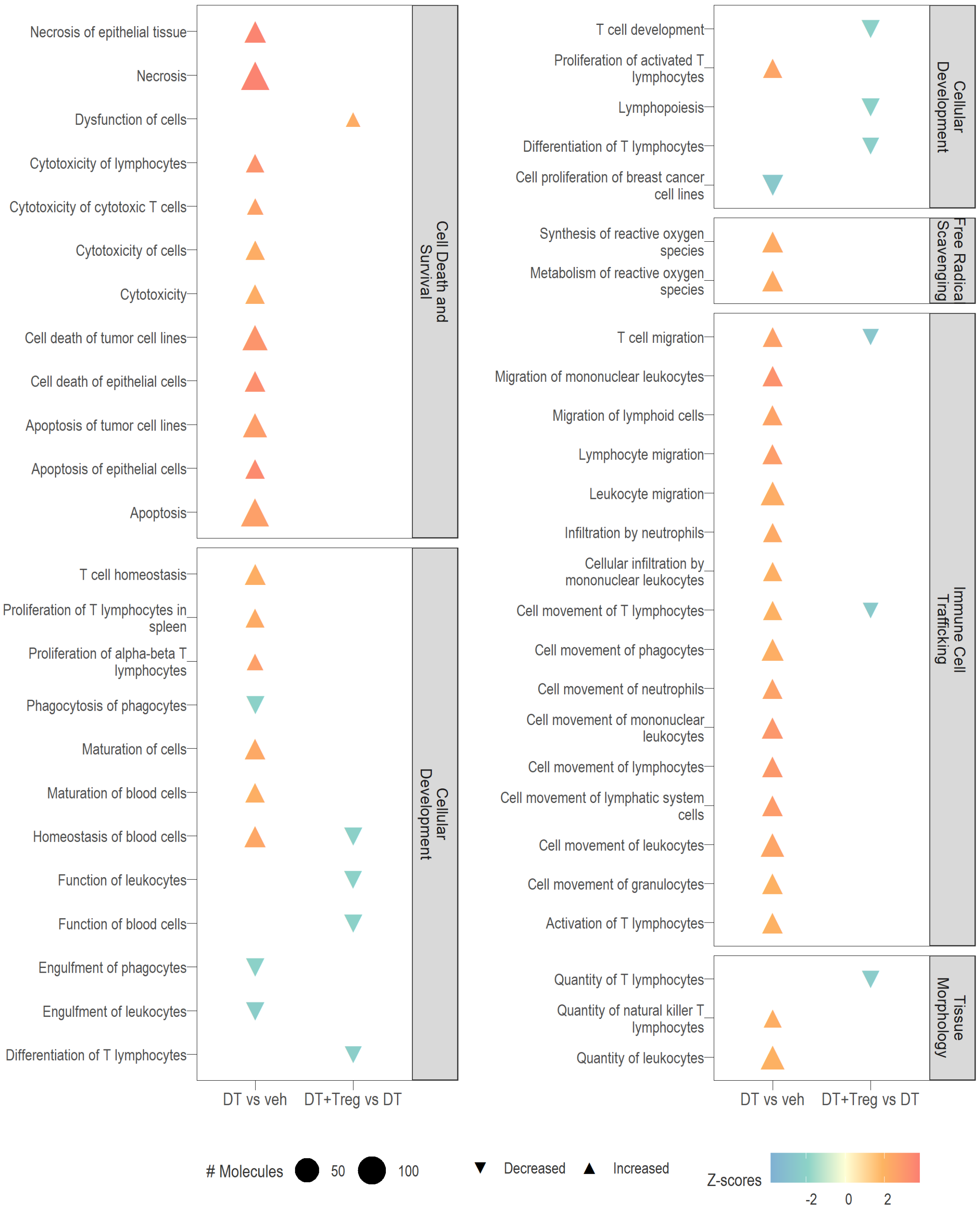

Supplemental Figure 9: Ingenuity Pathway Analysis (IPA) biological function and diseases. Pregnant *Foxp3*^{DTR} mice were administered PBS (veh; N=6) or DT (N=5) i.p. on GD3.5 and GD5.5, and tissues collected on GD10.5. Some mice (N=5) also received Treg cells isolated from wild-type pregnant mice on GD2.5 and GD4.5. Significant DEGs (FDR < 0.1) were analysed for associated biological functions and diseases. All functions are significant (*P* <0.05) with z-score indicating the predicted activation state. Symbol size is proportional to the number of significant DEGs in each pathway, and direction of arrow symbol represents the direction of change (increase or decrease).

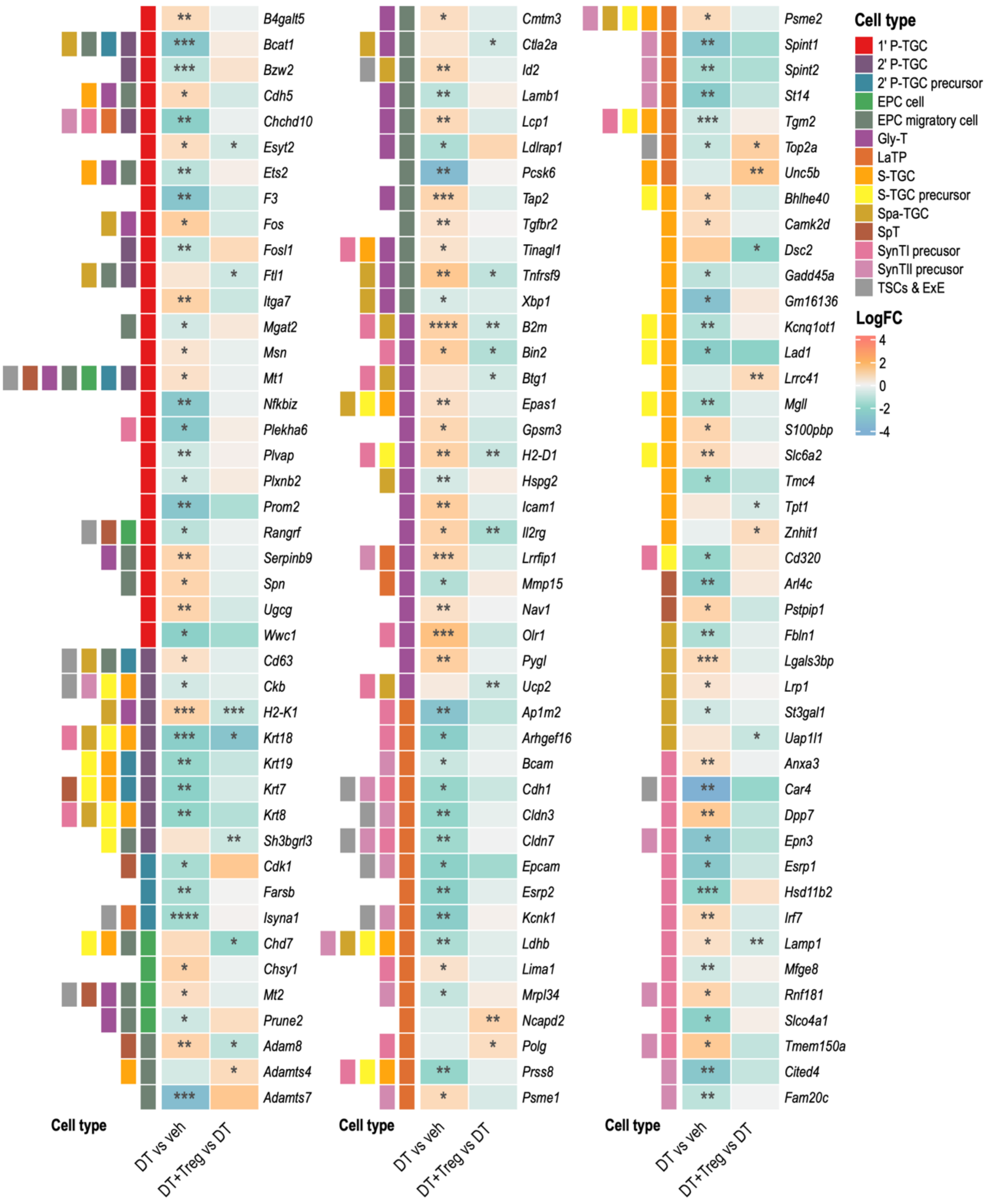

Supplemental Figure 10: Treg cell depletion causes a perturbation in decidual trophoblast genes in midgestation that is mitigated by Treg cell transfer. Pregnant *Foxp3*^{DTR} mice were administered PBS (veh; n=6) or DT (n=5) i.p. on GD3.5 and GD5.5, and decidual tissue was collected on GD10.5. Some mice (n=5) also received Treg cells isolated from wild-type pregnant mice on GD2.5 and GD4.5. Functional heatmap of significant DEGs (FDR <0.1) identified as indicative of altered extravillous trophoblasts based on reported expression in specific mouse trophoblast cell types (colour coded, LHS) (extracted from singe cell RNA-seq data reported in (24). Cell labels indicate the FDR-adjusted *P* value (FDR) of DEGs present in the RNA-seq dataset. *FDR <0.1; **FDR <0.05; ***FDR <0.01. 1’ P-TGC, primary parietal trophoblast giant cell; 2’ P-TGC, secondary parietal trophoblast giant cells; EPC, ectoplacental cone; Gly-T, glycogen trophoblast cells; LaTP, labyrinthine trophoblast; S-TGC, sinusoid trophoblast giant cell; Spa-TGC, spiral artery-associated trophoblast giant cell; SpT, spongiotrophoblast cell; SynT1 and SynTII, multinucleated syncytiotrophoblast cells; TSC, trophoblast stem cell; ExE, extraembryonic ectoderm.

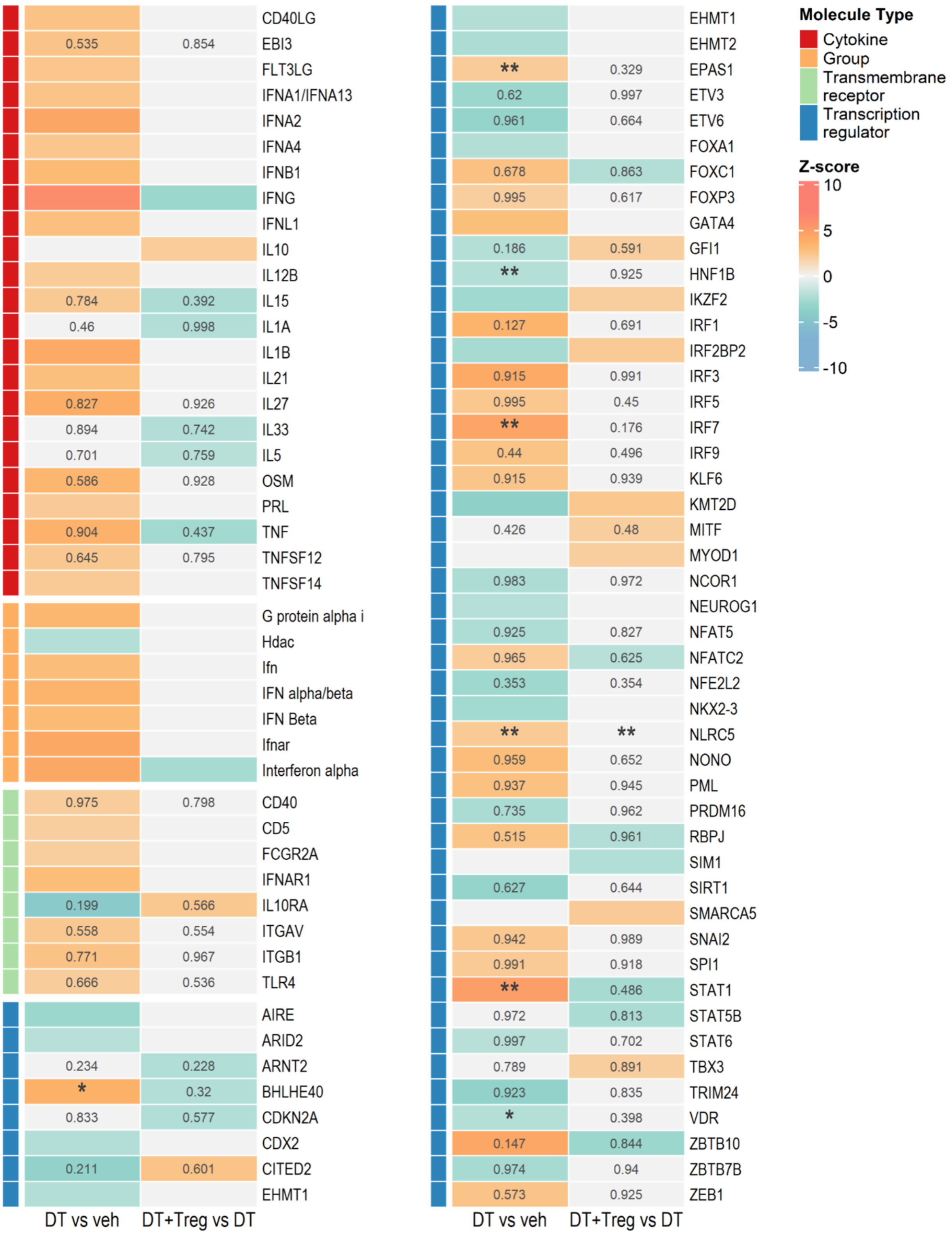

Supplemental Figure 11: Ingenuity Pathway Analysis (IPA) predicted upstream regulators. Pregnant *Foxp3*^{DTR} mice were administered PBS (veh; N=6) or DT (N=5) i.p. on GD3.5 and GD5.5, and tissues collected on GD10.5. Some mice (N=5) also received Treg cells isolated from wild-type pregnant mice on GD2.5 and GD4.5. Significant DEGs (FDR <0.1) were analysed for upstream regulators (*P* <0.05) that may explain the gene expression changes. Cell labels indicate the FDR-adjusted *P* value (FDR) of upstream regulators present in the RNA-seq dataset. *FDR <0.1; **FDR <0.05.

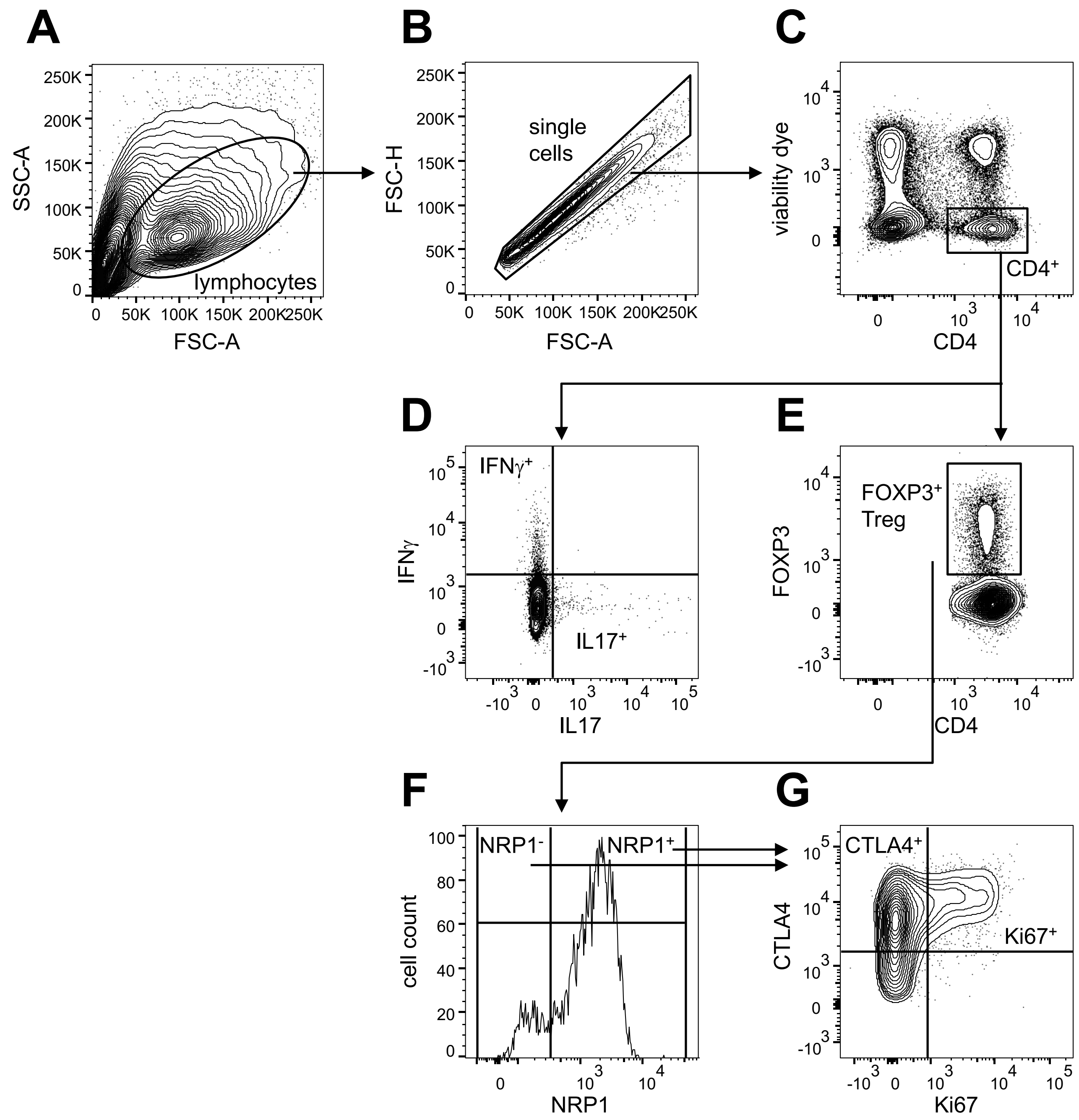

Supplemental Figure 12: Gating strategy to assess T cells. Pregnant *Foxp3*^{DTR} mice were administered PBS (veh) or DT i.p. on GD3.5 and GD5.5. Spleen, uDLN, uterus and/or decidua tissue were collected, processed into single cells and stained for flow cytometry analysis. (**A-B**) Side-scatter and forward-scatter gates were established to include singleton lymphocytes and exclude doublets and debris. (**C**) Viable CD4$^+$ lymphocytes were then identified. (**D**) IFNγ and IL17 expression was assessed within the total CD4$^+$ cell or FOXP3$^-$CD4$^+$ population. (**E**) Treg cells were identified within the CD4$^+$ T cell population based on expression of FOXP3, (**F**) with marker of suppressive competence CTLA4 then measured in NRP1$^-$ peripheral and NRP1$^+$ thymic Treg cells (**G**).

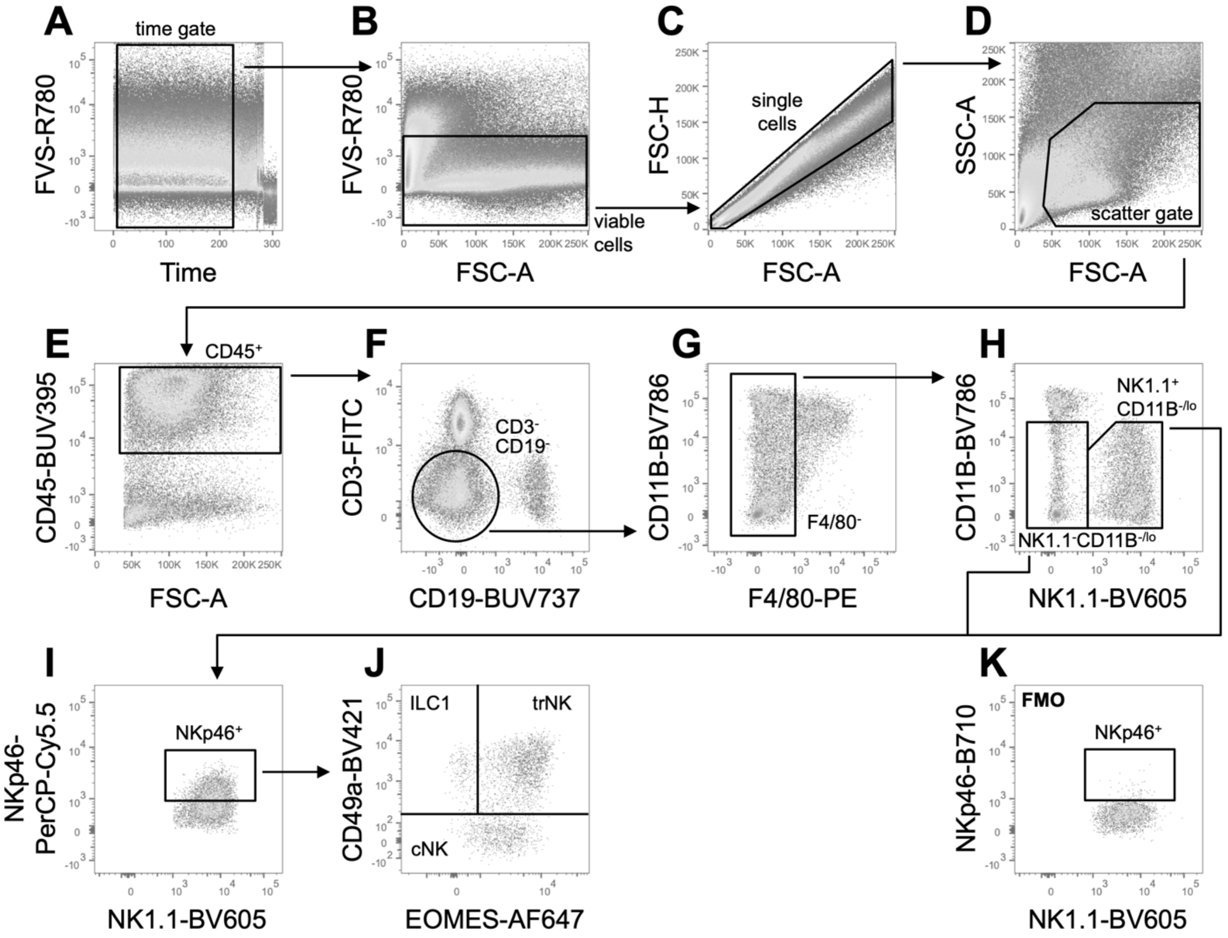

Supplemental Figure 13: Gating strategy for analysis of NK cell subsets. A time gate was used to eliminate periods of time during which events are not consistent (**A**). The time gate was placed on a viability gate (**B**), and a series of forward scatter (FCS) and side scatter (SSC) plots to include single cells and remove doublets (**C**), and exclude debris (**D**). Cells were then applied to a CD45 versus FCS-A plot to identify $CD45^+$ cells (**E**), from which $CD3^-CD19^-$ cells were identified to eliminate T and B cells respectively (**F**). Within the $CD3^-CD19^-$ population, $F4/80^-$ cells were identified (**G**), and then $NK1.1^+CD11B^{-/lo}$ NK cells or $NK1.1^-CD11B^{-/lo}$ cells were gated (**H**). Within the populations defined in (H), the $NKp46^+$ population was gated (**I**). Within the $NKp46^+NK1.1^+CD11B^-$/lo NK cells, CD49a and EOMES were assessed to define $CD49a^+EOMES^+$ tissue resident NK cells (trNK), $CD49a^-$ conventional NK cells (cNK), and $CD49a^+EOMES^-$ type 1 innate lymphoid cells (ILC1) (**J**). Gates were established based on unlabeled cells and fluorescent minus one (FMO) controls. The FMO control for NKp46 is shown (**K**). FVS; Fixable viability stain.

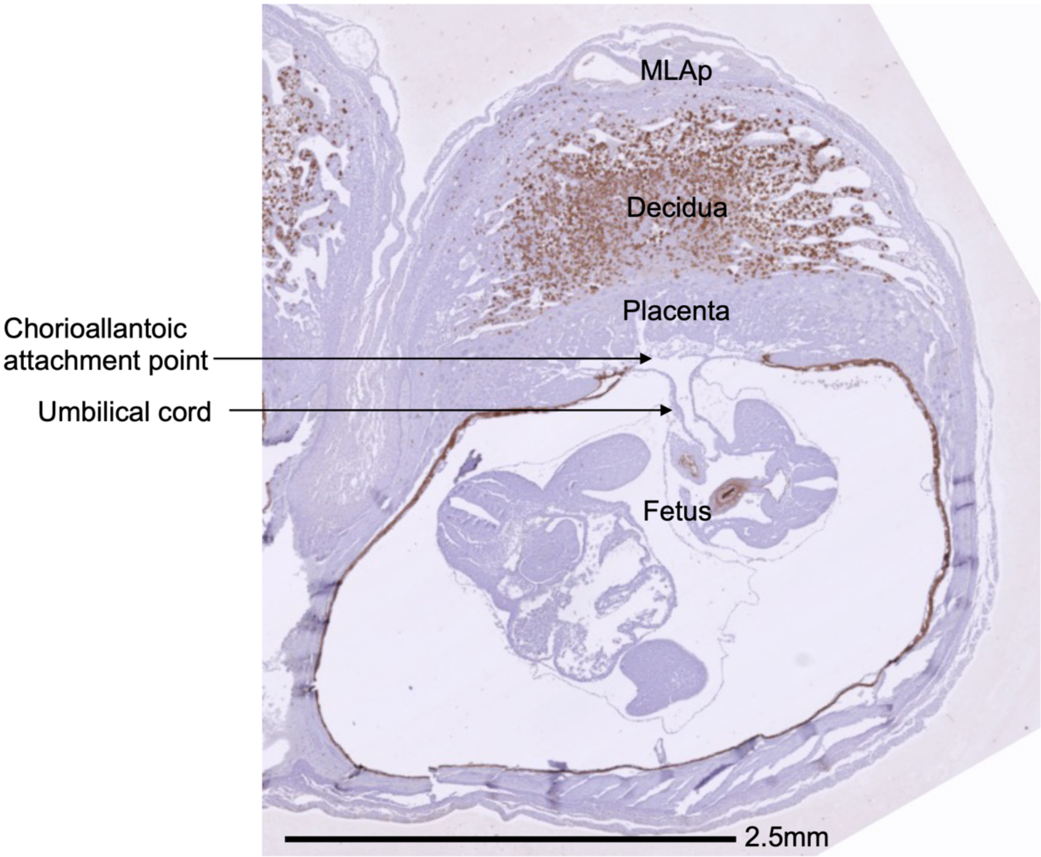

Supplemental Figure 14: Mid-sagittal sections of mouse implantation site. On GD10.5, serial sections of implantation sites were cut and stained to identify those closest to the mid-sagittal point, visualized by the presence of the chorioallantoic attachment tissue linking the fetus to the placenta. MLAp; mesometrial lymphoid aggregate of pregnancy. Scale bar; 2.5 mm.

REFERENCES

1. Kim JM, Rasmussen JP, and Rudensky AY. Regulatory T cells prevent catastrophic autoimmunity throughout the lifespan of mice. *Nat Immunol.* 2007;8(2):191-7.
2. Care AS, Bourque SL, Morton JS, Hjartarson EP, Robertson SA, and Davidge ST. Reduction in Regulatory T Cells in Early Pregnancy Causes Uterine Artery Dysfunction in Mice. *Hypertension.* 2018;72(1):177-87.
3. Schjenken JE, Moldenhauer LM, Zhang B, Care AS, Groome HM, Chan HY, et al. MicroRNA miR-155 is required for expansion of regulatory T cells to mediate robust pregnancy tolerance in mice. *Mucosal Immunol.* 2020.
4. Kieckbusch J, Gaynor LM, and Colucci F. Assessment of Maternal Vascular Remodeling During Pregnancy in the Mouse Uterus. *J Vis Exp.* 2015(106):e53534.
5. Wilson RL, Phillips JA, Bianco-Miotto T, McAninch D, Goh Z, Anderson PH, and Roberts CT. Reduced Dietary Calcium and Vitamin D Results in Preterm Birth and Altered Placental Morphogenesis in Mice During Pregnancy. *Reproductive Sciences.* 2020;27(6):1330-9.
6. Dilworth M, Kusinski L, Cowley E, Ward B, Husain S, Constancia M, et al. Placental-specific Igf2 knockout mice exhibit hypocalcemia and adaptive changes in placental calcium transport. *Proceedings of the National Academy of Sciences.* 2010;107(8):3894-9.
7. Renshall LJ, Morgan HL, Moens H, Cansfield D, Finn-Sell SL, Tropea T, et al. Melatonin Increases Fetal Weight in Wild-Type Mice but Not in Mouse Models of Fetal Growth Restriction. *Front Physiol.* 2018;9:1141.
8. Thiele K, Hierweger AM, Riquelme JIA, Solano ME, Lydon JP, and Arck PC. Impaired Progesterone-Responsiveness of CD11c(+) Dendritic Cells Affects the Generation of CD4(+) Regulatory T Cells and Is Associated With Intrauterine Growth Restriction in Mice. *Front Endocrinol (Lausanne).* 2019;10:96.
9. Kieffer TE, Chin PY, Green ES, Moldenhauer LM, Prins JR, and Robertson SA. Prednisolone in early pregnancy inhibits regulatory T cell generation and alters fetal and placental development in mice. *Molecular Human Reproduction.* 2020;26(5):340-52.
10. Kaur H, Wilson RL, Care AS, Muhlhausler BS, Roberts CT, and Gatford KL. Validation studies of a fluorescent method to measure placental glucose transport in mice. *Placenta.* 2019;76:23-9.
11. Chen Z, Zhang J, Hatta K, Lima PD, Yadi H, Colucci F, et al. DBA-lectin reactivity defines mouse uterine natural killer cell subsets with biased gene expression. *Biol Reprod.* 2012;87(4):81.
12. Chan HY, Moldenhauer LM, Groome HM, Schjenken JE, and Robertson SA. Toll-like receptor-4 null mutation causes fetal loss and fetal growth restriction associated with impaired maternal immune tolerance in mice. *Scientific Reports.* 2021;11(1):16569.
13. Schjenken JE, Sharkey DJ, Green ES, Chan HY, Matias RA, Moldenhauer LM, and Robertson SA. Sperm modulate uterine immune parameters relevant to embryo implantation and reproductive success in mice. *Communications Biology.* 2021;4(1):572.
14. Livak KJ, and Schmittgen TD. Analysis of relative gene expression data using real-time quantitative PCR and the 2(-Delta Delta C(T)) Method. *Methods.* 2001;25(4):402-8.
15. Andrews S. FastQC: a quality control tool for high throughput sequence data. Available online at: http://www.bioinformatics.babraham.ac.uk/projects/fastqc.
16. Martin M. Cutadapt removes adapter sequences from high-throughput sequencing reads. *2011.* 2011;17(1):3.
17. Dobin A, Davis CA, Schlesinger F, Drenkow J, Zaleski C, Jha S, et al. STAR: ultrafast universal RNA-seq aligner. *Bioinformatics.* 2013;29(1):15-21.

18. Chen Y, Lun A, and Smyth G. From reads to genes to pathways: differential expression analysis of RNA-Seq experiments using Rsubread and the edgeR quasi-likelihood pipeline [version 2; peer review: 5 approved]. *F1000Research.* 2016;5(1438).
19. Law CW, Chen Y, Shi W, and Smyth GK. voom: Precision weights unlock linear model analysis tools for RNA-seq read counts. *Genome Biol.* 2014;15(2):R29.
20. Wu T, Hu E, Xu S, Chen M, Guo P, Dai Z, et al. clusterProfiler 4.0: A universal enrichment tool for interpreting omics data. *The Innovation.* 2021;2(3).
21. Ashburner M, Ball CA, Blake JA, Botstein D, Butler H, Cherry JM, et al. Gene ontology: tool for the unification of biology. The Gene Ontology Consortium. *Nat Genet.* 2000;25(1):25-9.
22. Kanehisa M, Furumichi M, Sato Y, Kawashima M, and Ishiguro-Watanabe M. KEGG for taxonomy-based analysis of pathways and genomes. *Nucleic Acids Res.* 2023;51(D1):D587-d92.
23. Milacic M, Beavers D, Conley P, Gong C, Gillespie M, Griss J, et al. The Reactome Pathway Knowledgebase 2024. *Nucleic Acids Res.* 2024;52(D1):D672-d8.
24. Jiang X, Wang Y, Xiao Z, Yan L, Guo S, Wang Y, et al. A differentiation roadmap of murine placentation at single-cell resolution. *Cell Discov.* 2023;9(1):30.
